# Supplementary material for: Distinct genetic profiles influence body mass index between infancy and adolescence
Source: Nat Commun. 2026 Feb 19;17:1594. doi: 10.1038/s41467-026-69310-6 (PMC12920650; doi:10.1038/s41467-026-69310-6)
Supplement: Supplementary file 1 — Supplementary Information [file 41467_2026_69310_MOESM1_ESM.pdf]

Distinct genetic profiles influence body mass index between infancy and adolescence

Supplementary Materials

Table of Contents

Supplementary Materials 1

Supplementary Figures 5

Supplementary Figure 1: The fitted slopes (black line) of 16 randomly selected individuals with their observed BMI measurements from 1 to 18 years (black dots). 5

Supplementary Figure 2: The fitted slopes of 16 randomly selected individuals from 1 to 18 years (grey lines) and the population mean (red line)..6

Supplementary Figure 3: Estimates of genetic correlation (r_g) of BMI between different ages. 7

Supplementary Figure 4: Estimates of phenotypic correlation (r_p) of BMI between different ages...10

Supplementary Figure 5: Proportion of the total variance explained by PC 1-3 after eigenvalue decomposition of the genetic variance-covariance matrix (K_g). 10

Supplementary Figure 6: Mean body mass index (BMI) from one to 18 years of age for low, average or high polygenic score (PGS) of principal component one (PC1) of childhood genetic variance. 11

Supplementary Figure 7: Mean body mass index (BMI) from one to 18 years of age for three clusters based on a polygenic score (PGS) of principal component two (PC2). 12

Supplementary Figure 8: Estimated fixed effects of the polygenic score (PGS) of adult BMI on log(BMI) at different ages. 13

Supplementary Figure 9: SNP-based heritability and genetic variances with and without adjusting for polygenic score of adult body mass index (BMI).....................................14

Supplementary Figure 10: Quantile–quantile (QQ) plots of GWAS P-values for the five growth trajectory phenotypes; intercept (A), linear slope (B), quadratic polynomial (C), PC1 (D) and PC2 (E). 15

Supplementary Figure 11: Manhattan plots of GWAS for the five growth trajectory phenotypes; intercept (A), linear slope (B), quadratic polynomial (C), PC1 (D) and PC2 (E). 18

Supplementary Figure 12: Genome-wide genetic correlation between principal components of the BMI trajectory and a range of traits and diseases in later life. 19

Supplementary Figure 13: Comparison of SNP-based genetic correlations between BMI at different ages across early life, estimated using different modelling approaches in ALSPAC (random regression model using longitudinal data) and MoBa[1] (linkage disequilibrium score regression using cross-sectional data). 20

Supplementary Figure 14: Comparison of the SNP-based genetic correlation patterns between BMI at different ages across early life in ALSPAC using the random regression model (RRM) and cross-sectional genome-based restricted maximum likelihood (GREML) using GCTA. 21

Supplementary Figure 15: Unique individual variances, additive genetic variances, and SNP-based heritability between homogeneous and heterogeneous error variance models 22

Supplementary Figure 16: Principal components 1 and 2 of additive genetic effects on BMI for male and female twins in CODATwins project evaluated from 1 to 18 years of age 23

Supplementary Tables 24

Supplementary Table 1: Statistics for the models with different order of polynomials in random effect terms for additive genetics. 24

Supplementary Table 2: Estimates of fixed effects from the random regression model. 25

Supplementary Table 3: Variance-covariance matrices for random effect terms in the random regression model. 26

Supplementary Table 4: Estimated variance components and heritability of BMI at yearly intervals from one to 18 years of age from the random regression model 27

Supplementary Table 5: Estimated genetic correlations between BMI at yearly intervals from one to 18 years from the random regression model. 28

Supplementary Table 6: Estimated phenotypic correlations between BMI at yearly intervals from one to 18 years from the random regression model. 29

Supplementary Table 7: Estimated fixed effects from the random regression model with and without adjusting for adult BMI PGS. 30

Supplementary Table 8: Estimated variances and covariances for random effect terms in the random regression model for adult BMI PGS. 31

Supplementary Table 9: Estimated heritability of BMI at yearly intervals from one to 18 years of age from the random regression model conditioning on adult BMI PGS..........32

Supplementary Table 10: Estimated heritability from LDSC of derived phenotypes from random regression model..33

Supplementary Table 11: Estimated genetic correlations from LDSC across derived phenotypes from random regression model..33

Supplementary Table 12: Comparison of the SNP-based heritability between BMI at different ages across early life in ALSPAC using the random regression model (RRM), cross-sectional genome-based restricted maximum likelihood (GREML) using GCTA, and MoBa (linkage disequilibrium score regression using cross-sectional data)............35

Supplementary Table 13: Estimated genetic correlations between multiple ages from cross-sectional genetic analyses in GCTA 36

Supplementary Table 14: Estimated fixed effects from the random regression model with heterogenous errors...37

Supplementary Table 15: Estimated variances and covariances for random effect terms in the random regression model heterogeneous errors 38

Supplementary Table 16: Estimated heritability of BMI at yearly intervals from one to 18 years of age from the random regression model with heterogeneous errors 40

Supplementary Table 17 Descriptive statistics of the final dataset in ALSPAC cohort used for RRM analysis..41

Supplementary Notes 43

Supplementary Note 1: Validation and model checking..43

Supplementary Note 2: ASReml .as file for main analysis..44

Supplementary Note 3: R script for calculating 95% confidence intervals for the eigenvalues using numerical simulation 45

Supplementary Note 4: ASReml .as file for adjusting for adult BMI PGS 47

Supplementary Note 5: ASReml .as file for heterogeneity analysis...............................48

Supplementary Note 6: Eigenvalue decomposition of genetic correlation matrix of BMI from the previously published COADTwins project 50

Supplementary Figures

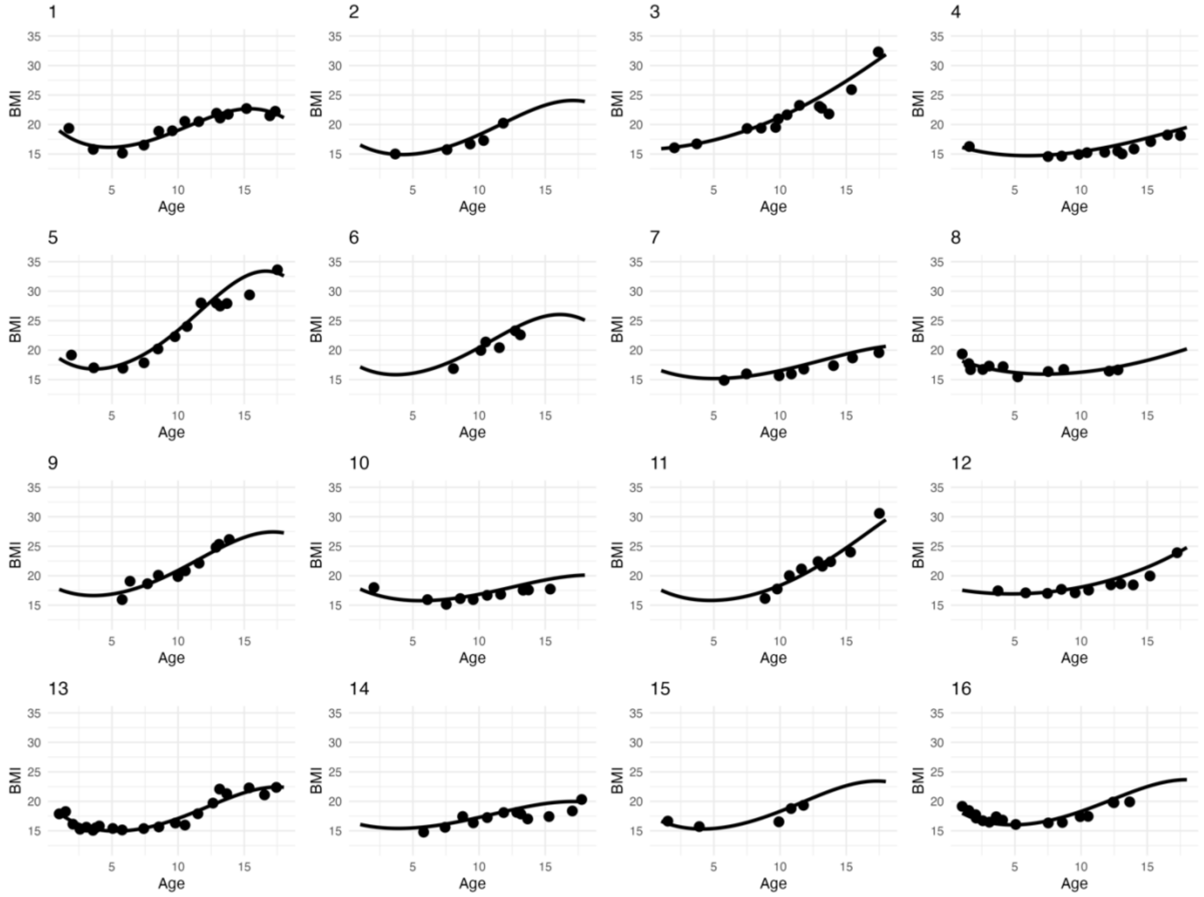

Supplementary Figure 1: The fitted slopes (black line) of 16 randomly selected individuals with their observed BMI measurements from 1 to 18 years (black dots).

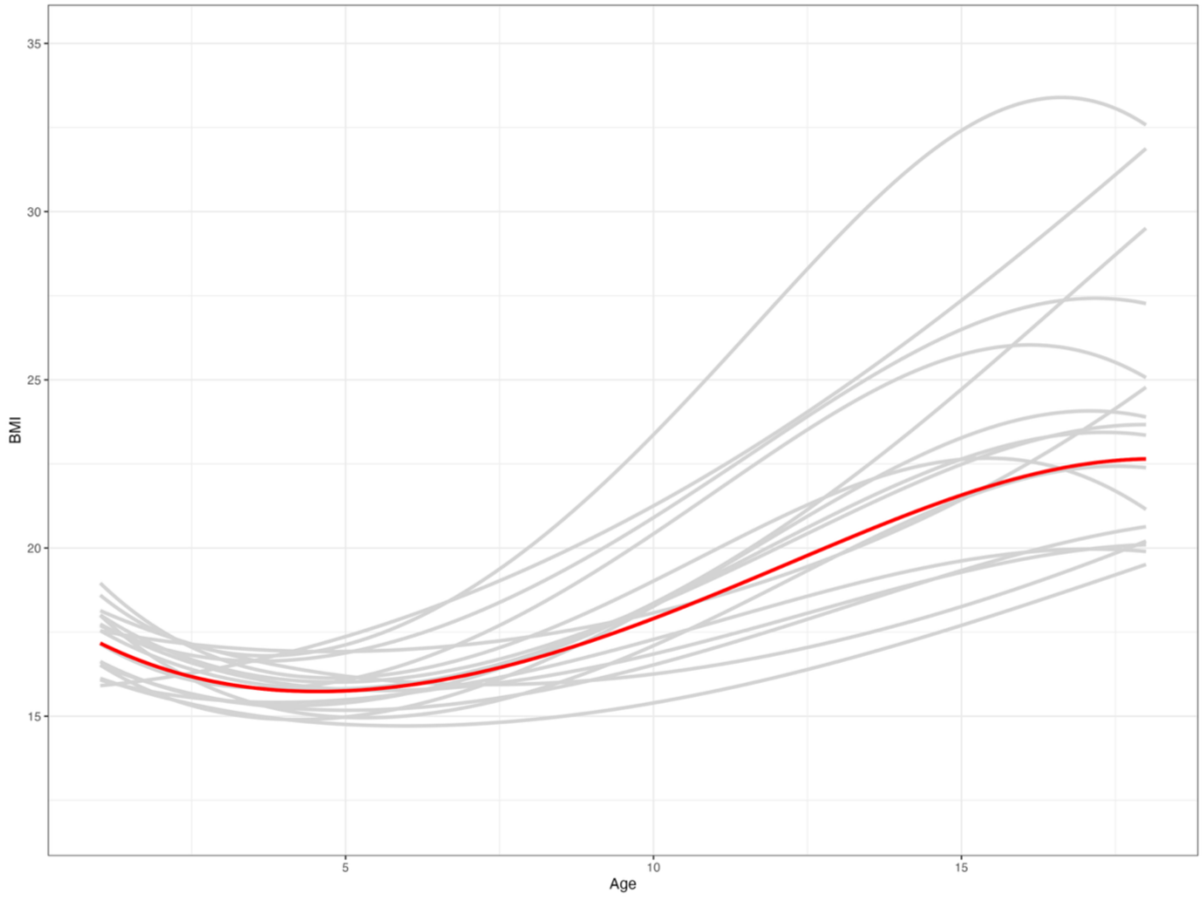

Supplementary Figure 2: The fitted slopes of 16 randomly selected individuals from 1 to 18 years (grey lines) and the population mean (red line).

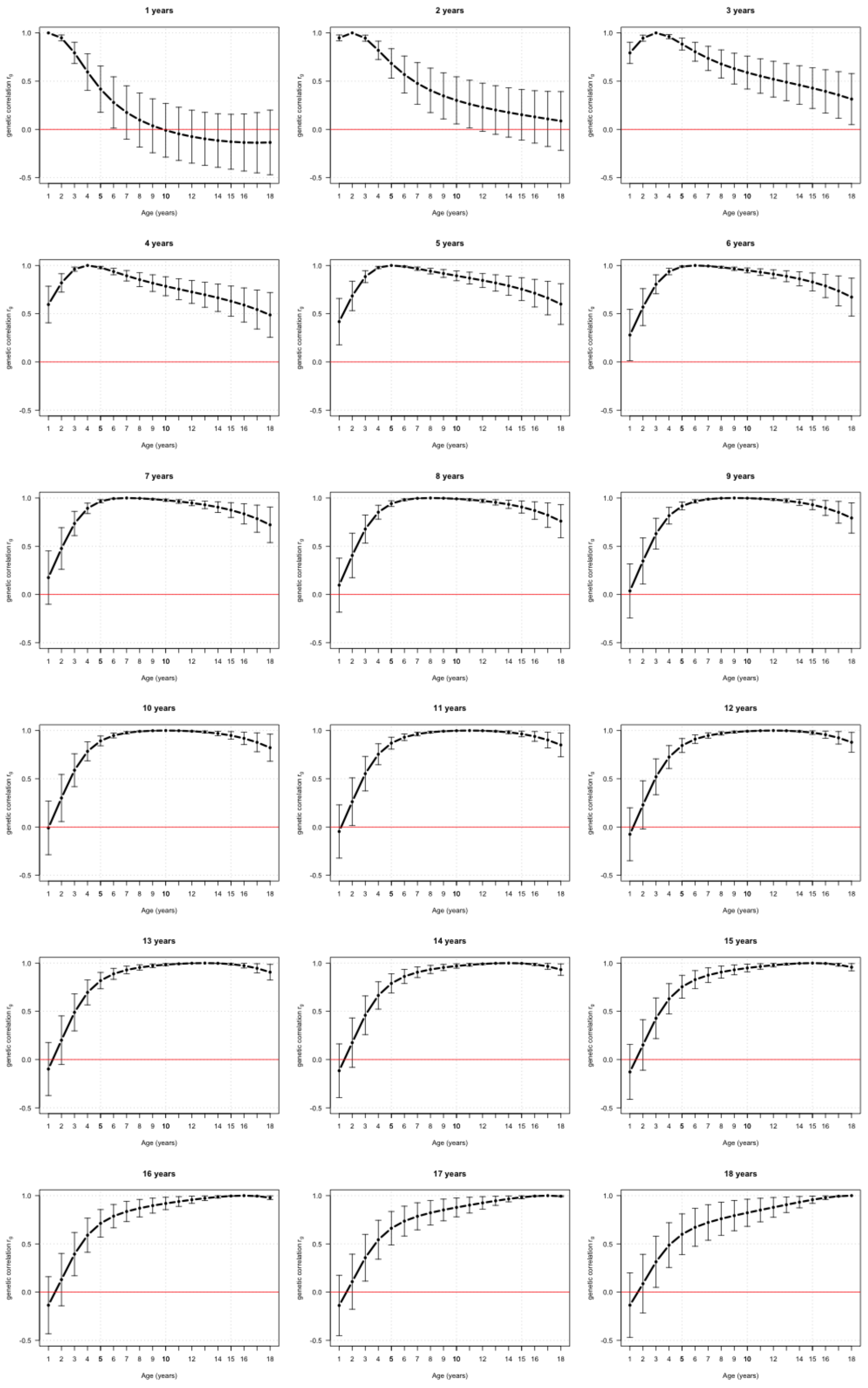

Supplementary Figure 3: Estimates of genetic correlation (*r_g*) of BMI between different ages.

Each plot represents the genetic correlations (represented along the y-axis) for a single age (given in the plot title) with all ages between 1 to 18 years in 1-year intervals (represented along the x-axis). Error bars represent the 95% confidence intervals. The red horizontal line indicates

when genetic correlation is zero. The sample size is 6, 291 ALSPAC participants with 65,930 repeated BMI measurements. Source data are provided as a Source Data file.

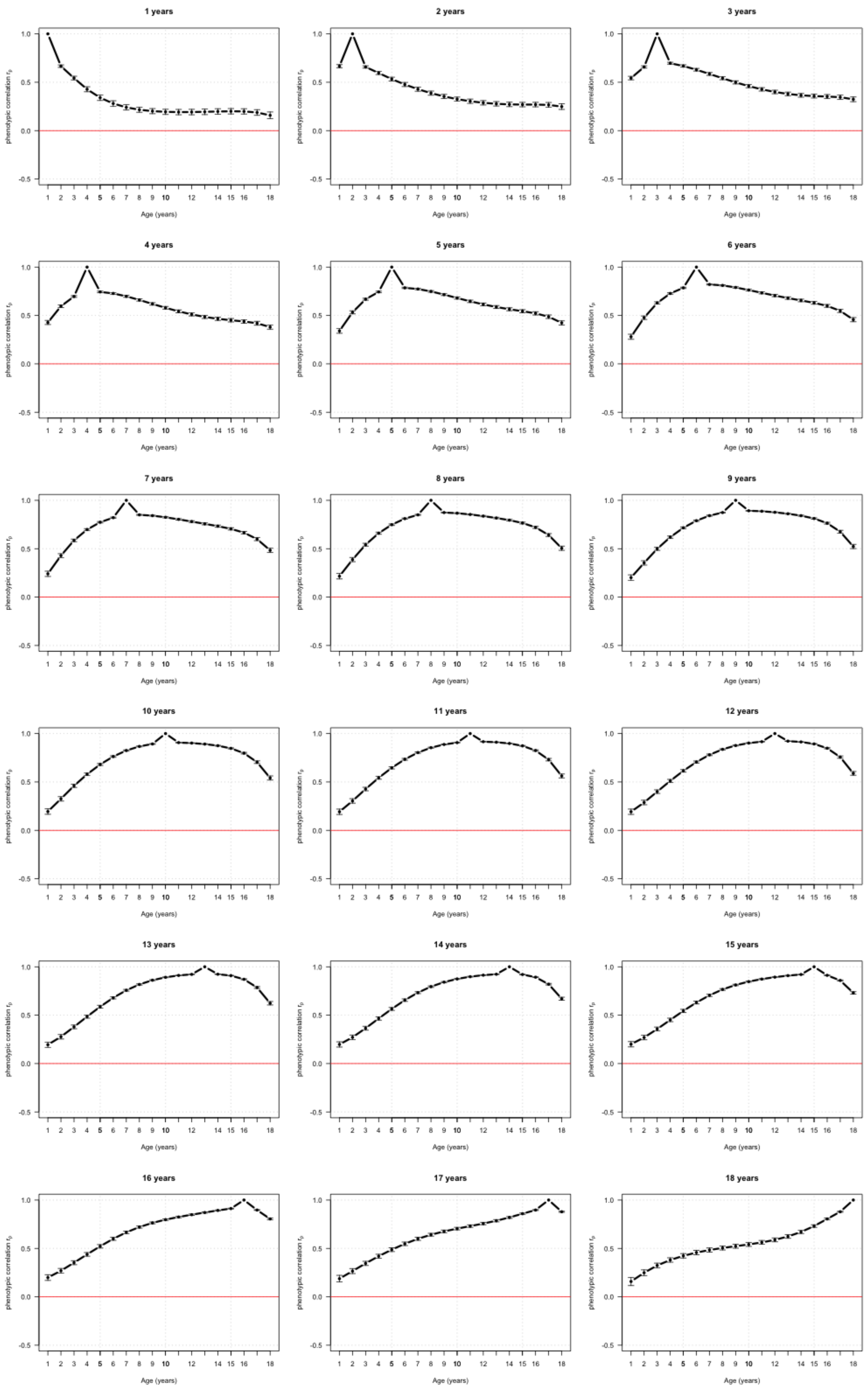
1 years
2 years
3 years
4 years
5 years
6 years
7 years
8 years
9 years
10 years
11 years
12 years
13 years
14 years
15 years
16 years
17 years
18 years
phenotypic correlation r_p
Age (years)

Supplementary Figure 4: Estimates of phenotypic correlation (r_p) of BMI between different ages.

Each plot represents the phenotypic correlations (y-axis) for a single age (given in the plot title) with all ages between 1 to 18 years in 1-year intervals (x-axis). Error bars represent the 95% confidence intervals. The red horizontal line indicates when phenotypic is zero. The sample size is 6,291 ALSPAC participants with 65,930 repeated BMI measurements. Source data are provided as a Source Data file.

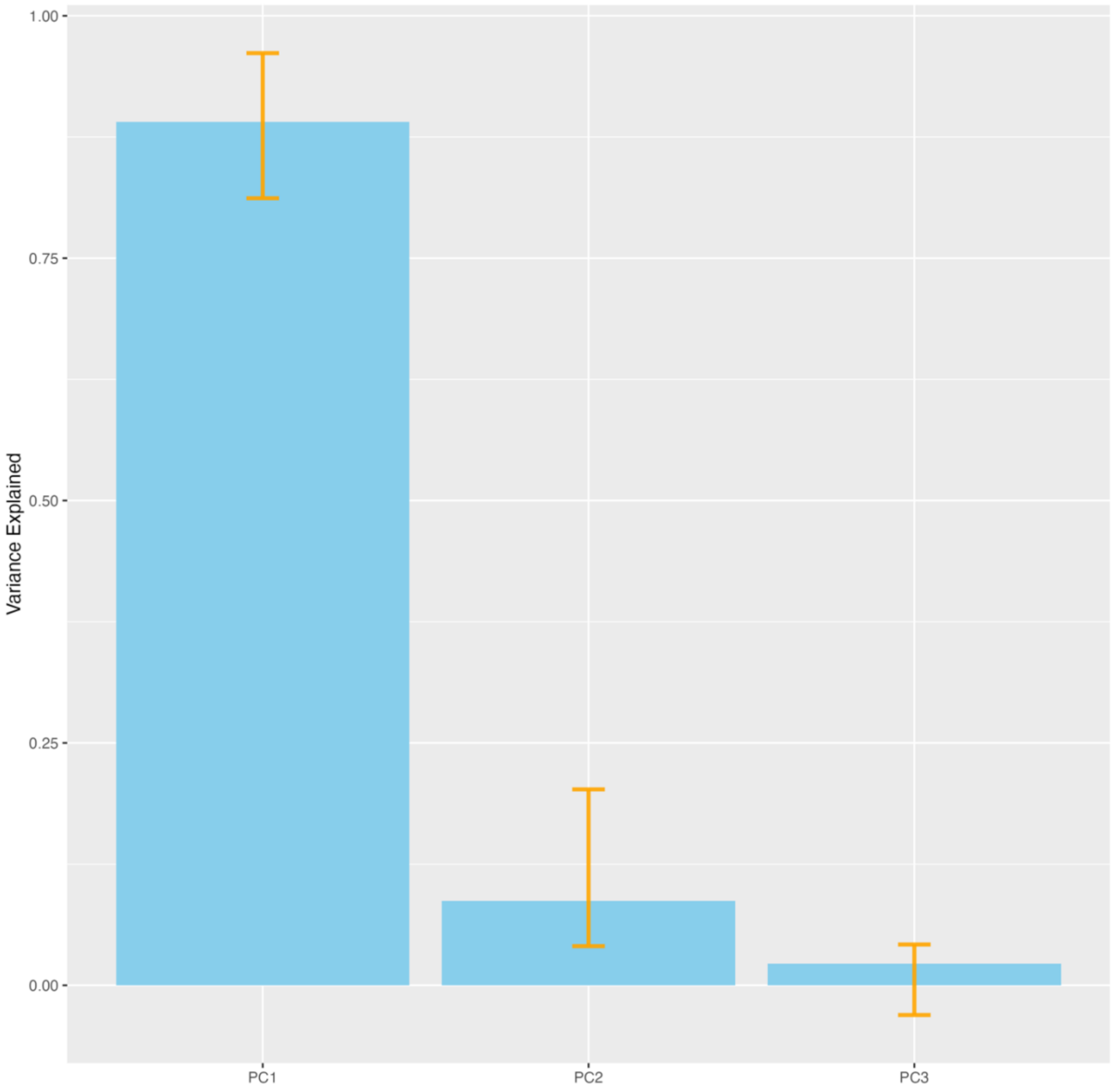

Supplementary Figure 5: **Proportion of the total variance explained by PC 1-3 after eigenvalue decomposition of the genetic variance-covariance matrix (K_g).**

The orange error bars are 95% confidence intervals calculated by numerical simulation.

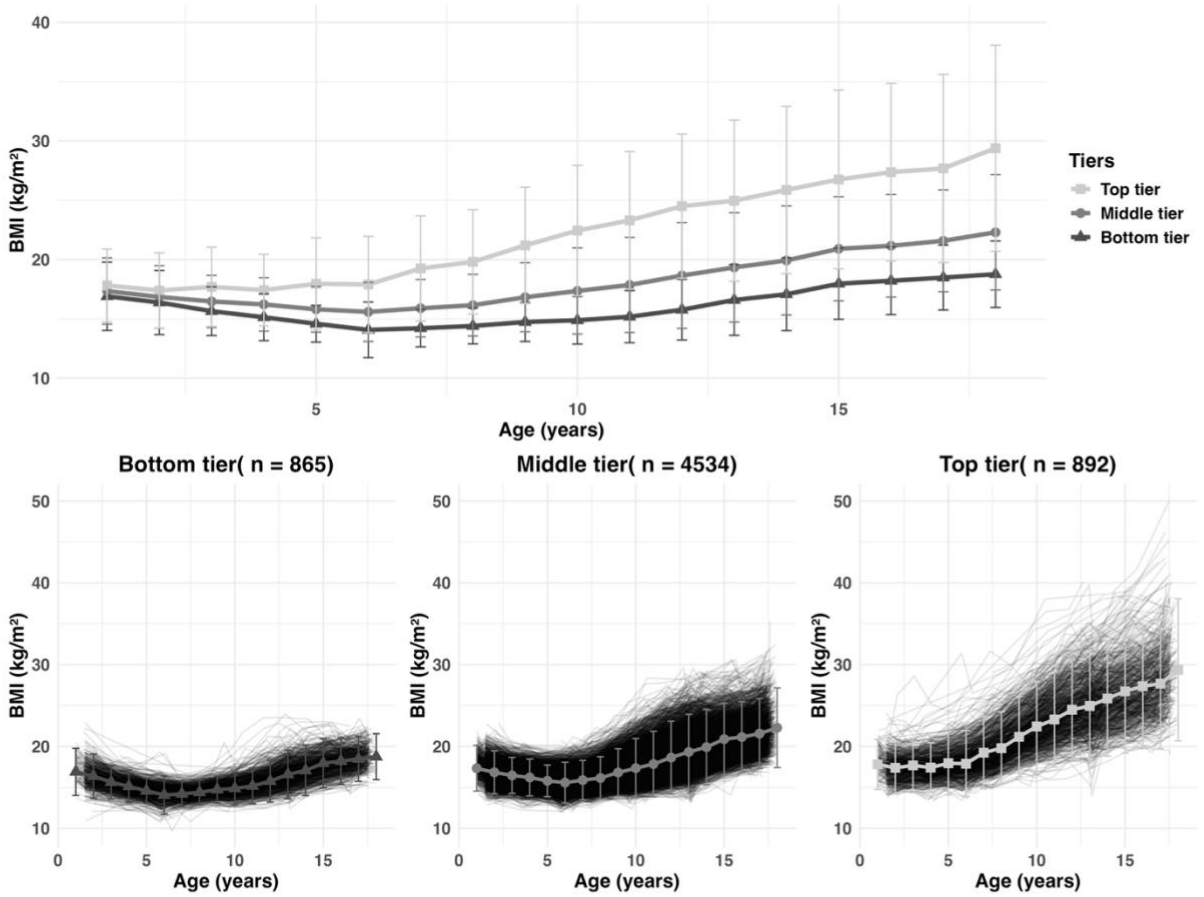

Supplementary Figure 6: Mean body mass index (BMI) from one to 18 years of age for low, average or high polygenic score (PGS) of principal component one (PC1) of childhood genetic variance.

The upper plot illustrates the mean BMI (points with error bars represent the 95% confidence intervals) from age one to 18 years for each of the three clusters classified by a PGS of PC1: the top tier are those individuals greater than one standard deviation higher than the mean PGS (N=892), the middle tier are those individuals within one standard deviation of the mean PGS (N=4,534), and the bottom tier are those individuals greater than one standard deviation lower than the mean PGS(N=865). The lower plots display the same mean BMI as in the upper plot from one to 18 years for the cluster (grey lines) and BMI trajectories for each individual within the three clusters (black lines). Source data are provided as a Source Data file.

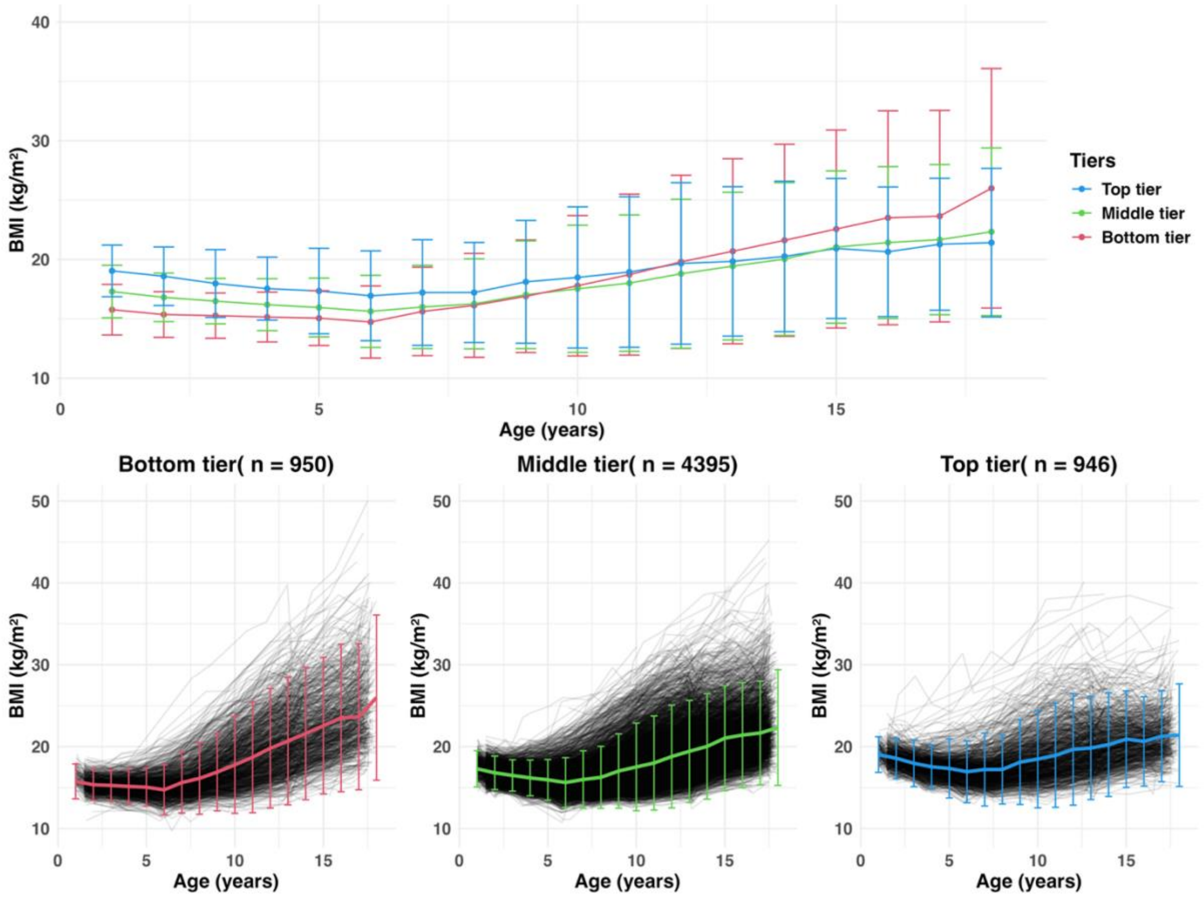

Supplementary Figure 7: Mean body mass index (BMI) from one to 18 years of age for three clusters based on a polygenic score (PGS) of principal component two (PC2).

The upper plot illustrates the mean BMI of each of the three clusters classified by PGS of PC2 (middle tier [individuals within one standard deviation of the mean PGS], top tier, and bottom tier of the residual) across yearly age bins ranging from 1 to 18 years. The lower plots separately display the mean BMI of each yearly age bin (colored lines) and individual trajectories for these three clusters (black lines). The sample size is 6,291 ALSPAC participants with 65,930 repeated BMI measurements. Source data are provided as a Source Data file.

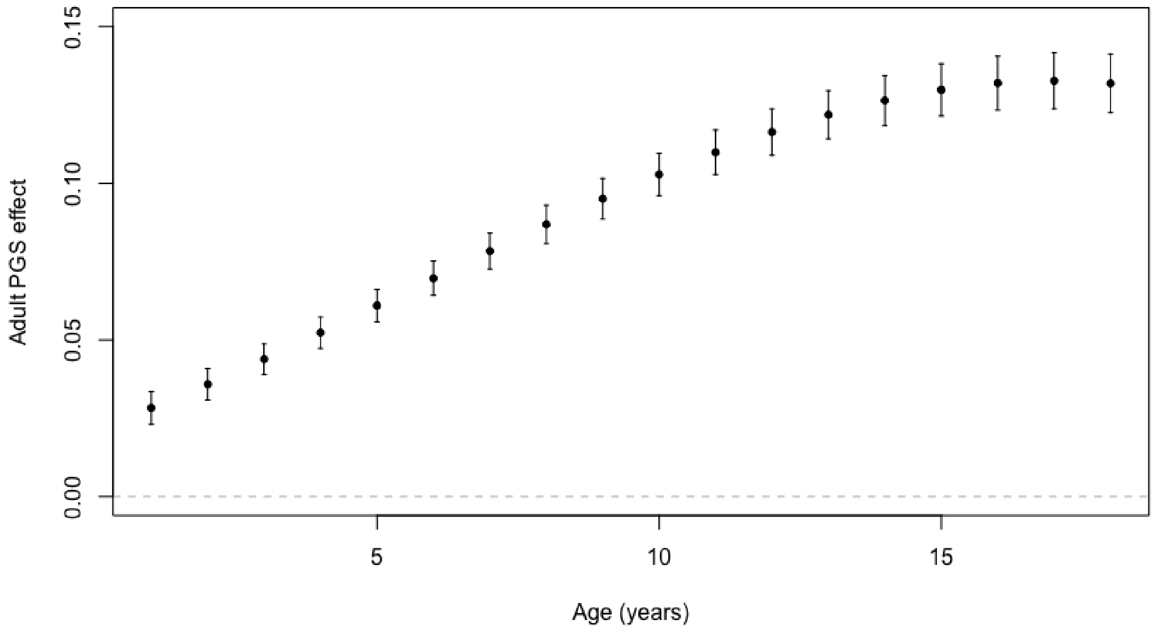

Supplementary Figure 8: Estimated fixed effects of the polygenic score (PGS) of adult BMI on log(BMI) at different ages.

The error bars are 95% confidence intervals calculated by the ASREML output of the prediction variance matrix (.vrb file). The mean log-transformed BMI in the studied cohort is 2.89, with a standard deviation of 0.17. The sample size is 6,291 ALSPAC participants with 65,930 repeated BMI measurements. Source data are provided as a Source Data file.

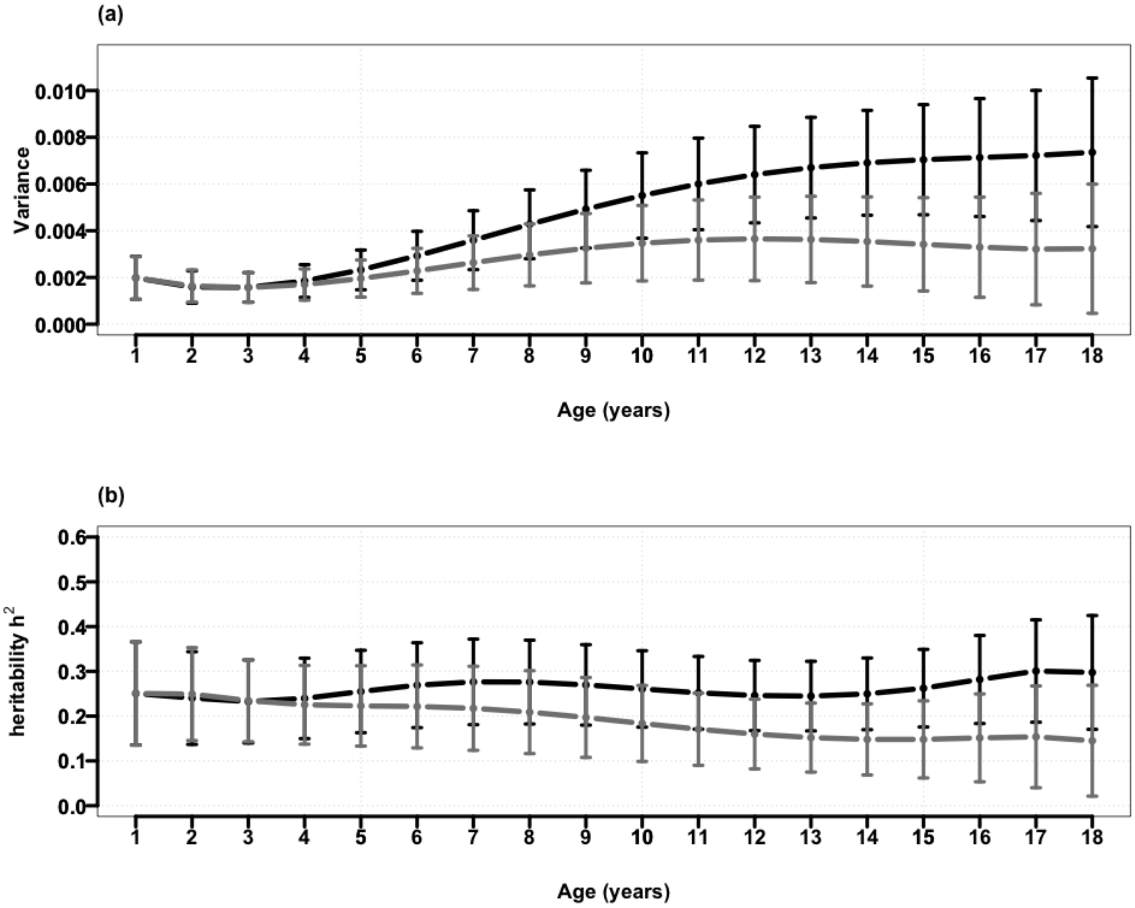

Supplementary Figure 9: SNP-based heritability and genetic variances with and without adjusting for polygenic score of adult body mass index (BMI).

Points indicate (a) additive genetic variance by age , and (b) heritability estimates of BMI, with (grey) and without (black) adjusting for a polygenic score (PGS) of adult BMI in the fixed effects of the RRM. The analyses include 6,291 participants. Error bars represent 95% confidence intervals. Source data are provided as a Source Data file.

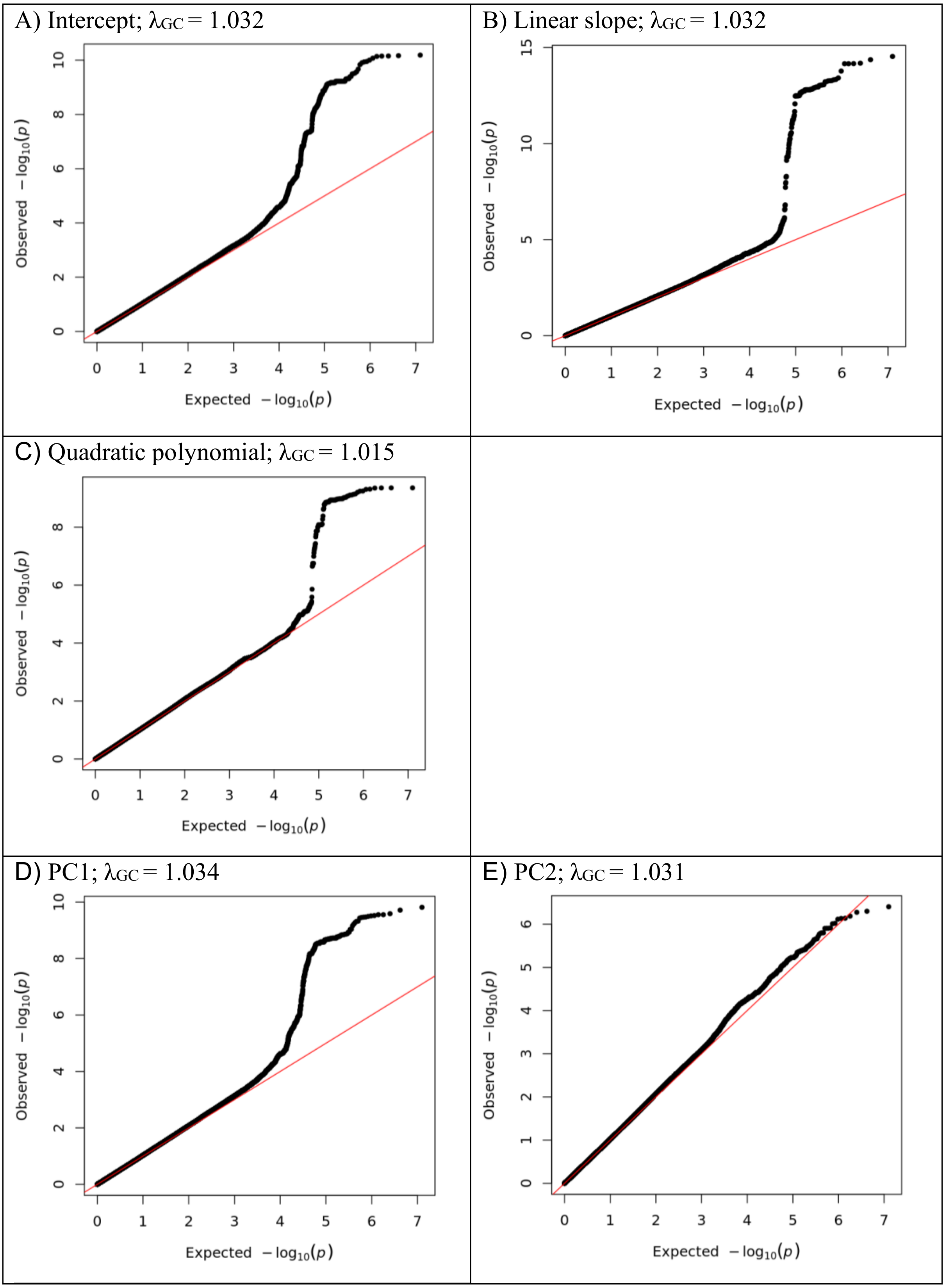

Supplementary Figure 10: Quantile–quantile (QQ) plots of GWAS P-values for the five growth trajectory phenotypes; intercept (A), linear slope (B), quadratic polynomial (C), PC1 (D) and PC2 (E).

QQ plots of GWAS (n=6,291) comparing observed versus expected $-\log_{10}(P)$ values under the null hypothesis for the growth trajectory phenotypes. Genomic inflation factors (λ_{GC}) are also reported above the plot.

A) Intercept

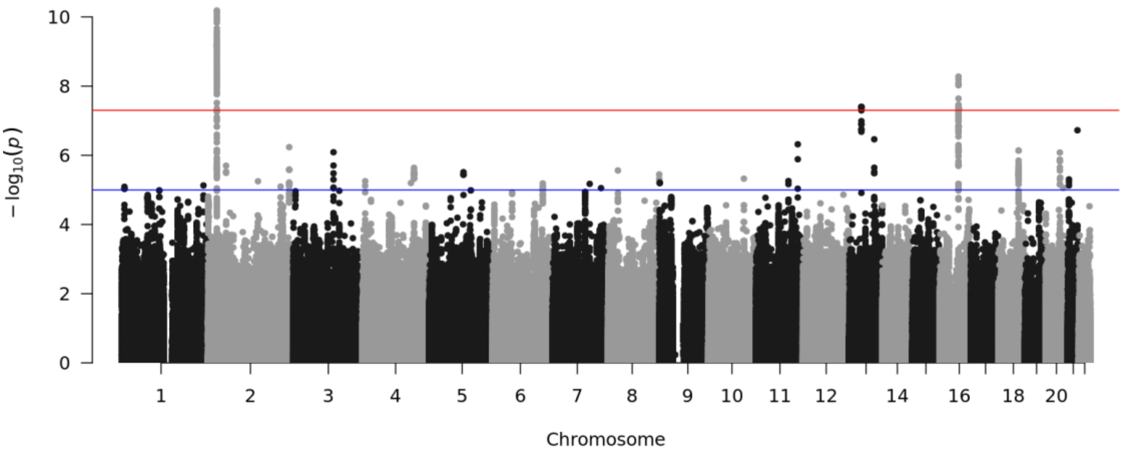

B) Linear slope

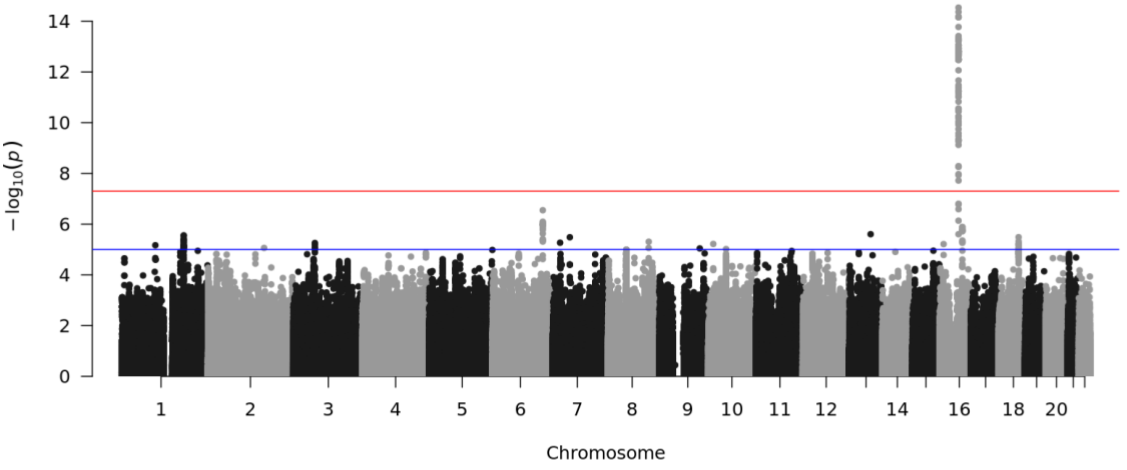

C) Quadratic polynomial

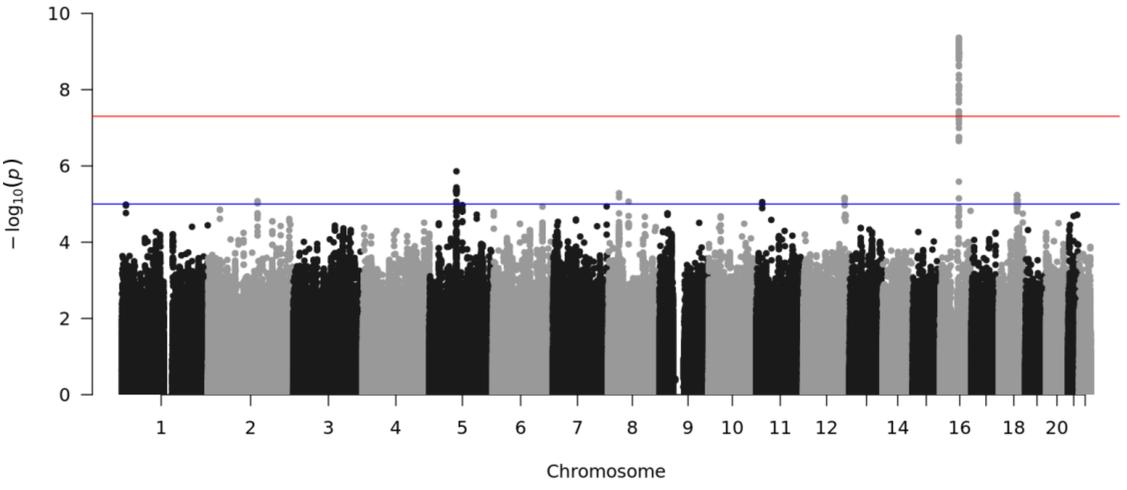

D) PC1

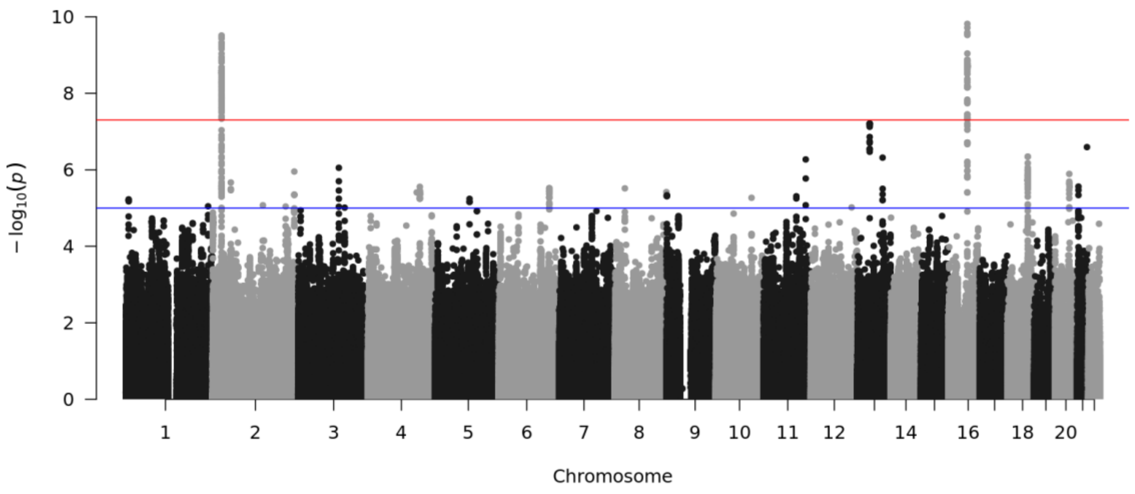

E) PC2

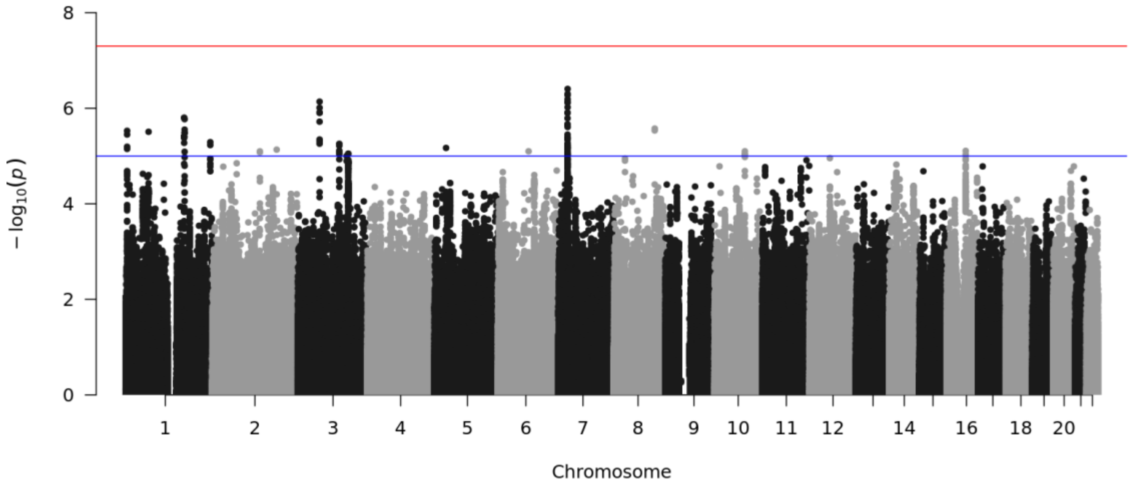

Supplementary Figure 11: Manhattan plots of GWAS for the five growth trajectory phenotypes; intercept (A), linear slope (B), quadratic polynomial (C), PC1 (D) and PC2 (E).

The two-sided association P-value on the $-\log_{10}$ scale obtained from the GWAS (n= 6,291) for each of the SNPs (y-axis) was plotted against the genomic position (NCBI Build 37; x-axis). The red line corresponds to the genome-wide significance level of $P<5x10^{-8}$, which accounts for multiple testing, and the blue line indicates a suggestive threshold ($P = 1 \times 10^{-5}$).

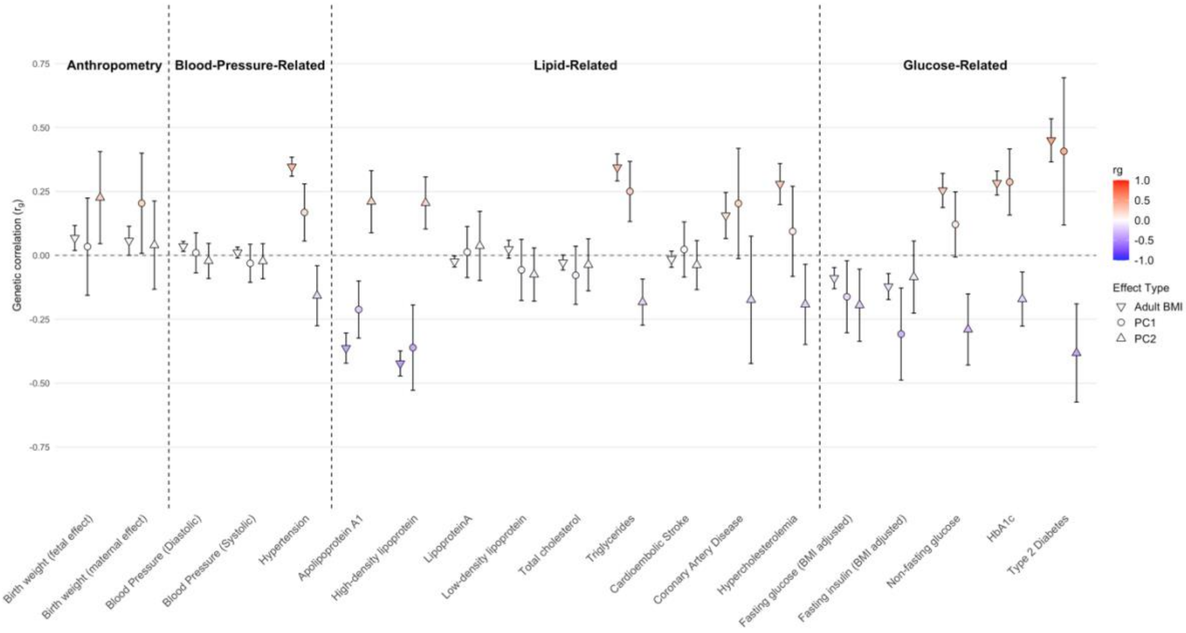

Supplementary Figure 12: Genome-wide genetic correlation between principal components of the BMI trajectory and a range of traits and diseases in later life. Genetic correlation (r_g) between the top two principal components of the BMI trajectory and the traits, and corresponding 95% CIs, were estimated using linkage disequilibrium score regression. r_g between adult BMI and the selected traits is presented as a reference. The genetic correlation estimates are colour-coded according to their intensity and direction (red, positive correlation; blue, negative correlation). See Supplementary Data 1 for the references for each of the traits displayed, as well as the genetic correlation results for other traits.

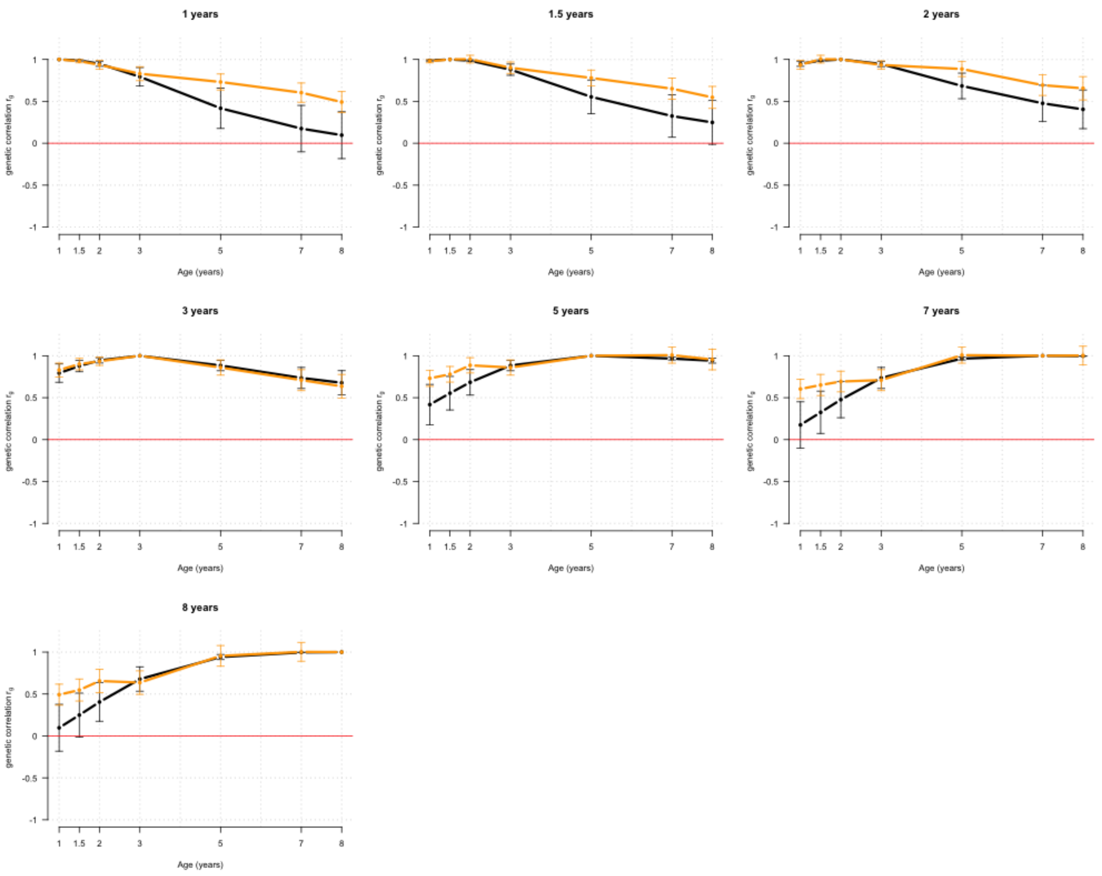

Supplementary Figure 13: Comparison of SNP-based genetic correlations between BMI at different ages across early life, estimated using different modelling approaches in ALSPAC (random regression model using longitudinal data) and MoBa[1] (linkage disequilibrium score regression using cross-sectional data).

Each panel shows the genetic correlation between BMI at a given target age (title of each subplot) and BMI at other ages. Black lines represent estimates from the random regression model (RRM) in ALSPAC; yellow lines represent estimates from cross-sectional GWAS in MoBa using LD Score Regression (LDSC). Error bars indicate 95% confidence intervals. Note that BMI measurements before age 1 were excluded in ALSPAC but included in the MoBa phenotype definition at year 1, which may contribute to differences in early estimates. Despite differences in data structure and modelling, both approaches show higher genetic correlations between temporally adjacent timepoints. The sample size is 6,291 ALSPAC participants with 65,930 repeated BMI measurements. Source data are provided as a Source Data file.

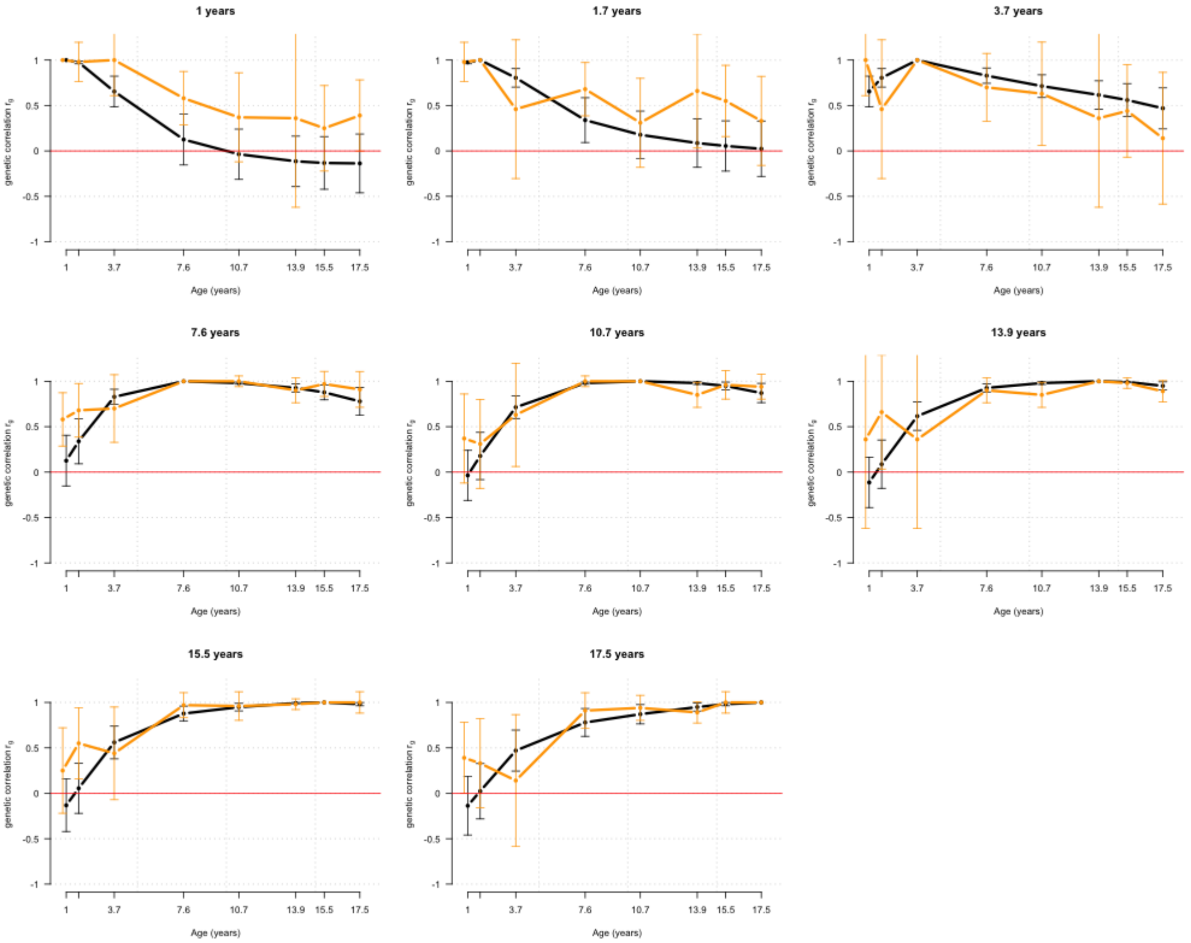

Supplementary Figure 14: Comparison of the SNP-based genetic correlation patterns between BMI at different ages across early life in ALSPAC using the random regression model (RRM) and cross-sectional genome-based restricted maximum likelihood (GREML) using GCTA.
Each panel displays the SNP-based genetic correlation between BMI at a given target age (title of each subplot) and BMI at other ages. Black lines represent estimates from the RRM applied to longitudinal ALSPAC data. Yellow lines represent estimates based on cross-sectional analyses using GCTA. Error bars indicate 95% confidence intervals. ALSPAC lacks dense follow-up data around 1 year of age, and thus, the 0.8-year cross-sectional estimate was used as a proxy. The sample size is 6,291 ALSPAC participants with 65,930 repeated BMI measurements. Source data are provided as a Source Data file.

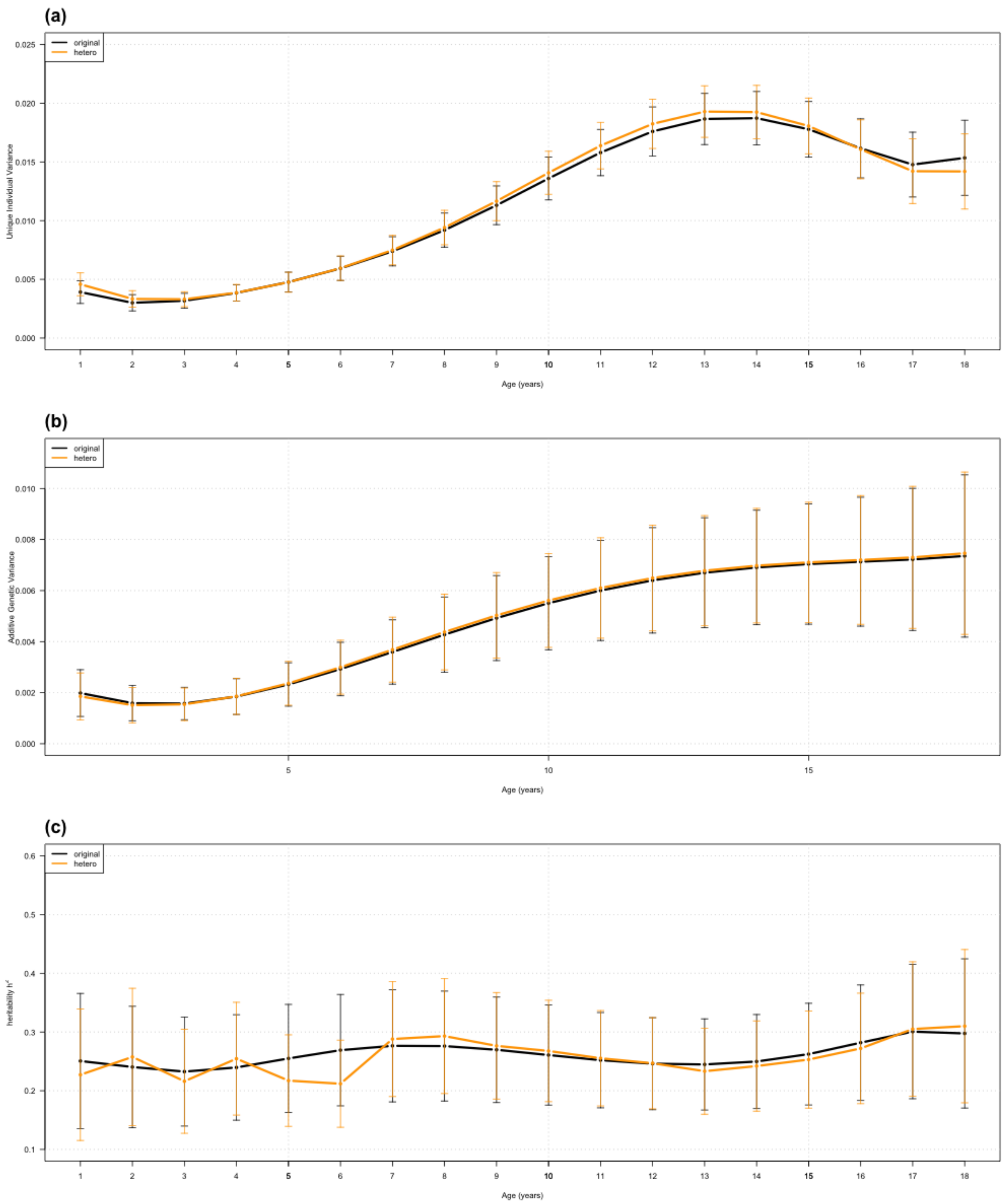

Supplementary Figure 15: Unique individual variances, additive genetic variances, and SNP-based heritability between homogeneous and heterogeneous error variance models

The upper plot (a) presents the change of variances of unique individual effects as age increases using homogeneous (black) and heterogeneous (yellow) error variance models; the middle plot (b) presents the change of variances of additive genetic effects as age increases using homogeneous (black) and heterogeneous (yellow) error variance models; the lower plot (c) shows the estimated SNP-based heritability of BMI from 1 to 18 years of age using homogeneous (black) and heterogeneous (yellow) error variance models. Original: homogeneous error variance model; hetero: heterogeneous error variance model. The sample

size is 6,291 ALSPAC participants with 65,930 repeated BMI measurements. Source data are provided as a Source Data file.

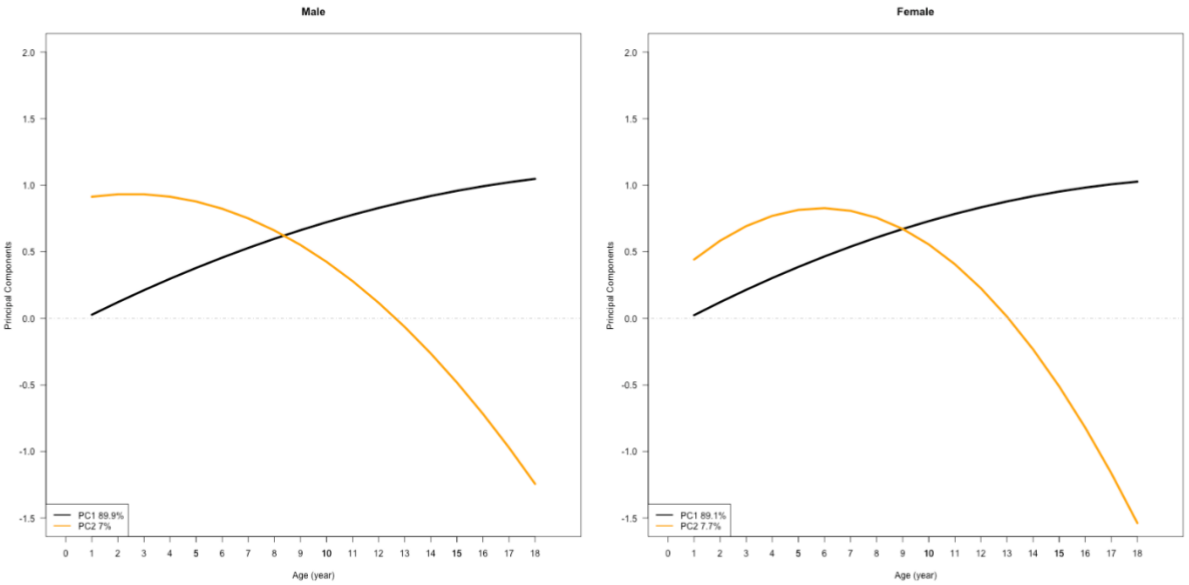

Supplementary Figure 16: Principal components 1 and 2 of additive genetic effects on BMI for male and female twins in CODATwins project evaluated from 1 to 18 years of age

This figure illustrates the principal components (PC) representing the additive genetic effects on BMI as age changes in male (left) and female (right) twins in the CODATwins project. The x-axis denotes age in years, while the y-axis is the value of the eigenfunction. PC1 and PC2 are represented in lines in different colours (black and orange, respectively). Each line depicts the changing genetic effects of the PC on BMI across different ages.

Supplementary Tables

Supplementary Table 1: Statistics for the models with different order of polynomials in random effect terms for additive genetics.

Order of polynomial	n	AIC	LogL	P
**Intercept**	12	-293986.62	147005.31	-
**Intercept, age**	14	-294012.80	147020.40	8.80×10^{-5}
**Intercept, age, age^2,**	17	-294024.55	147029.27	4.97×10^{-4}
**Intercept, age, age^2, age^3**	21	-294016.61	147029.31	0.999

LogL: log-likelihood; P: P-value of likelihood Number of parameters estimated (n), Alkaline Information Criterion (AIC) and log-likeihood (LogL) for the models with different orders of polynomials for additive genetics effects in the random regression model. Also shown are P-values from log-likelihood ratio tests between models with n vs. n-1 order polynomials. Statistical significance was assessed using a two-sided χ^2 test (1 degree of freedom). No multiple-comparison correction was applied.

Supplementary Table 2: Estimates of fixed effects from the random regression model.

Model Term	Level	Fixed effects	SE	*P value*
Intercept	-	2.9020	0.0019	0
Legendre polynomials	slope	0.1619	0.0014	0
	$slope^2$	0.0535	0.0009	0
	$slope^3$	-0.0344	0.0007	0
Source(clinic/questionnaire)	questionnaire	-0.0171	0.0006	0
Sex(male/female)	female	-0.0111	0.0028	6.94E-05
Interaction between sex and Legendre polynomials	female*slope	-0.0207	0.0020	0
	$female*slope^2$	0.0093	0.0013	4.58E-12
	$female*slope^3$	0.0044	0.0009	3.31E-06

Source: measurement sources; SE: standard errors of estimated fixed effects. * P value = 0 stands for $< 2.225\times10^{-308}$. Statistical significance was assessed using a two-sided χ^2 test (1 degree of freedom). No multiple-comparison correction was applied.

Supplementary Table 3: Variance-covariance matrices for random effect terms in the random regression model.

**Unique Individual Variance-Covariance Matrix (K_i)**				
	intercept	slope	$slope^2$	$slope^3$
intercept	0.0165 (0.0012)			
slope	0.0053 (0.0005)	0.0035 (0.0003)		
$slope^2$	-0.0028 (0.0003)	-0.0006 (0.0001)	0.0017 (0.0000)	
$slope^3$	-0.0017 (0.0001)	-0.0010 (0.0000)	0.0005 (0.0000)	0.0008 (0.0000)
**Additive Genetic Variance-Covariance Matrix (K_g)**				
	intercept	slope	$slope^2$	
intercept	0.0073 (0.0013)			
slope	0.0024 (0.0005)	0.0017 (0.0003)		
$slope^2$	-0.0012 (0.0003)	-0.0004 (0.0001)	0.0004 (0.0001)	
**Residual Variance (σ_e^2)**				
0.0020 (0.0000)				

The variances (SE) are on the diagonals and the values below the diagonal are the covariances (SE)

Supplementary Table 4: **Estimated variance components and heritability of BMI at yearly intervals from one to 18 years of age from the random regression model**

Age (years)	V_g	Var(V_g)	V_i	Var(V_i)	V_P	Var(V_P)	σ_e^2	Var(σ_e^2)	h^2_{SNP}	SE(h^2_{SNP})
**1**	1.98×10^{-3}	2.19×10^{-7}	3.91×10^{-3}	2.44×10^{-7}	7.92×10^{-3}	4.78×10^{-8}	2.02×10^{-3}	1.88×10^{-10}	0.251	0.059
**2**	1.59×10^{-3}	1.24×10^{-7}	3.00×10^{-3}	1.25×10^{-7}	6.61×10^{-3}	1.46×10^{-8}	2.02×10^{-3}	1.88×10^{-10}	0.240	0.053
**3**	1.57×10^{-3}	1.05×10^{-7}	3.18×10^{-3}	1.04×10^{-7}	6.77×10^{-3}	1.12×10^{-8}	2.02×10^{-3}	1.88×10^{-10}	0.233	0.047
**4**	1.85×10^{-3}	1.28×10^{-7}	3.84×10^{-3}	1.26×10^{-7}	7.71×10^{-3}	1.46×10^{-8}	2.02×10^{-3}	1.88×10^{-10}	0.240	0.046
**5**	2.32×10^{-3}	1.88×10^{-7}	4.77×10^{-3}	1.84×10^{-7}	9.11×10^{-3}	2.12×10^{-8}	2.02×10^{-3}	1.88×10^{-10}	0.255	0.047
**6**	2.93×10^{-3}	2.85×10^{-7}	5.93×10^{-3}	2.77×10^{-7}	1.09×10^{-2}	3.12×10^{-8}	2.02×10^{-3}	1.88×10^{-10}	0.269	0.048
**7**	3.60×10^{-3}	4.14×10^{-7}	7.39×10^{-3}	4.02×10^{-7}	1.30×10^{-2}	4.60×10^{-8}	2.02×10^{-3}	1.88×10^{-10}	0.276	0.049
**8**	4.27×10^{-3}	5.64×10^{-7}	9.19×10^{-3}	5.50×10^{-7}	1.55×10^{-2}	6.62×10^{-8}	2.02×10^{-3}	1.88×10^{-10}	0.276	0.048
**9**	4.92×10^{-3}	7.21×10^{-7}	1.13×10^{-2}	7.09×10^{-7}	1.82×10^{-2}	9.21×10^{-8}	2.02×10^{-3}	1.88×10^{-10}	0.270	0.046
**10**	5.51×10^{-3}	8.70×10^{-7}	1.36×10^{-2}	8.65×10^{-7}	2.11×10^{-2}	1.22×10^{-7}	2.02×10^{-3}	1.88×10^{-10}	0.261	0.044
**11**	6.00×10^{-3}	1.00×10^{-6}	1.58×10^{-2}	1.01×10^{-6}	2.38×10^{-2}	1.55×10^{-7}	2.02×10^{-3}	1.88×10^{-10}	0.252	0.041
**12**	6.40×10^{-3}	1.11×10^{-6}	1.76×10^{-2}	1.13×10^{-6}	2.60×10^{-2}	1.87×10^{-7}	2.02×10^{-3}	1.88×10^{-10}	0.246	0.040
**13**	6.70×10^{-3}	1.21×10^{-6}	1.87×10^{-2}	1.24×10^{-6}	2.74×10^{-2}	2.13×10^{-7}	2.02×10^{-3}	1.88×10^{-10}	0.245	0.040
**14**	6.91×10^{-3}	1.31×10^{-6}	1.87×10^{-2}	1.34×10^{-6}	2.77×10^{-2}	2.26×10^{-7}	2.02×10^{-3}	1.88×10^{-10}	0.250	0.041
**15**	7.04×10^{-3}	1.45×10^{-6}	1.78×10^{-2}	1.46×10^{-6}	2.68×10^{-2}	2.24×10^{-7}	2.02×10^{-3}	1.88×10^{-10}	0.262	0.044
**16**	7.13×10^{-3}	1.66×10^{-6}	1.62×10^{-2}	1.64×10^{-6}	2.53×10^{-2}	2.10×10^{-7}	2.02×10^{-3}	1.88×10^{-10}	0.282	0.050
**17**	7.22×10^{-3}	2.02×10^{-6}	1.48×10^{-2}	1.98×10^{-6}	2.40×10^{-2}	2.18×10^{-7}	2.02×10^{-3}	1.88×10^{-10}	0.301	0.058
**18**	7.36×10^{-3}	2.63×10^{-6}	1.53×10^{-2}	2.67×10^{-6}	2.47×10^{-2}	3.39×10^{-7}	2.02×10^{-3}	1.88×10^{-10}	0.298	0.065

V_g: estimated additive genetic variance; Var(V_g): estimated variance of the additive genetic variance component (V_g); V_i: estimated unique individual variance; Var(V_i): estimated variance of unique individual variance component; V_P: estimated phenotypic variance; Var(V_P): estimated variance of phenotypic variance; σ_e^2: estimated residual variance; Var(σ_e^2): estimated variance of residual variance component. h^2_{snp}: estimated SNP-heritability; SE(h^2_{snp}): standard error of SNP-heritability estimate.

Supplementary Table 5: **Estimated genetic correlations between BMI at yearly intervals from one to 18 years from the random regression model.**

Age (years)	1	2	3	4	5	6	7	8	9	10	11	12	13	14	15	16	17	18
**1**	1																	
**2**	0.948 (0.015)	1																
**3**	0.793 (0.056)	0.945 (0.016)	1															
**4**	0.595 (0.097)	0.819 (0.049)	0.961 (0.011)	1														
**5**	0.418 (0.123)	0.684 (0.078)	0.884 (0.031)	0.979 (0.006)	1													
**6**	0.279 (0.136)	0.568 (0.098)	0.805 (0.05)	0.937 (0.017)	0.989 (0.003)	1												
**7**	0.175 (0.141)	0.477 (0.11)	0.736 (0.064)	0.893 (0.028)	0.966 (0.009)	0.994 (0.002)	1											
**8**	0.097 (0.143)	0.405 (0.118)	0.678 (0.074)	0.853 (0.037)	0.942 (0.015)	0.981 (0.005)	0.996 (0.001)	1										
**9**	0.037 (0.143)	0.347 (0.122)	0.63 (0.082)	0.817 (0.044)	0.917 (0.021)	0.966 (0.009)	0.988 (0.003)	0.998 (0.001)	1									
**10**	-0.009 (0.142)	0.301 (0.125)	0.589 (0.087)	0.784 (0.05)	0.893 (0.026)	0.949 (0.013)	0.977 (0.006)	0.992 (0.002)	0.998 (0.001)	1								
**11**	-0.046 (0.141)	0.262 (0.126)	0.553 (0.091)	0.754 (0.055)	0.869 (0.031)	0.931 (0.017)	0.964 (0.01)	0.983 (0.005)	0.993 (0.002)	0.998 (0.001)	1							
**12**	-0.075 (0.140)	0.229 (0.127)	0.521 (0.095)	0.725 (0.061)	0.845 (0.037)	0.911 (0.023)	0.948 (0.014)	0.971 (0.009)	0.984 (0.005)	0.993 (0.002)	0.998 (0.001)	1						
**13**	-0.098 (0.140)	0.201 (0.129)	0.49 (0.098)	0.696 (0.066)	0.819 (0.043)	0.888 (0.029)	0.929 (0.02)	0.955 (0.014)	0.972 (0.009)	0.984 (0.006)	0.993 (0.003)	0.998 (0.001)	1					
**14**	-0.115 (0.142)	0.175 (0.130)	0.459 (0.102)	0.665 (0.072)	0.789 (0.051)	0.861 (0.037)	0.905 (0.028)	0.934 (0.022)	0.954 (0.016)	0.97 (0.011)	0.982 (0.007)	0.991 (0.004)	0.998 (0.001)	1				
**15**	-0.128 (0.145)	0.152 (0.134)	0.428 (0.107)	0.630 (0.08)	0.755 (0.061)	0.829 (0.048)	0.875 (0.039)	0.907 (0.032)	0.93 (0.026)	0.949 (0.02)	0.965 (0.015)	0.978 (0.009)	0.989 (0.005)	0.997 (0.001)	1			
**16**	-0.136 (0.151)	0.130 (0.138)	0.394 (0.114)	0.59 (0.09)	0.713 (0.073)	0.788 (0.062)	0.836 (0.053)	0.87 (0.046)	0.897 (0.040)	0.919 (0.033)	0.939 (0.026)	0.957 (0.019)	0.973 (0.012)	0.987 (0.006)	0.996 (0.002)	1		
**17**	-0.138 (0.159)	0.108 (0.146)	0.356 (0.123)	0.543 (0.103)	0.662 (0.089)	0.736 (0.079)	0.786 (0.072)	0.822 (0.065)	0.852 (0.058)	0.878 (0.05)	0.902 (0.042)	0.925 (0.033)	0.946 (0.024)	0.966 (0.015)	0.983 (0.008)	0.995 (0.002)	1	
**18**	-0.135 (0.171)	0.087 (0.155)	0.314 (0.135)	0.487 (0.118)	0.600 (0.108)	0.672 (0.100)	0.722 (0.094)	0.76 (0.087)	0.793 (0.080)	0.822 (0.072)	0.851 (0.062)	0.879 (0.052)	0.906 (0.041)	0.933 (0.030)	0.957 (0.019)	0.979 (0.010)	0.994 (0.003)	1

The lower triangle shows the genetic correlations between BMI across different ages (in years) as indicated in the column and row headers. Standard errors for each estimate are given in brackets.

Supplementary Table 6: **Estimated phenotypic correlations between BMI at yearly intervals from one to 18 years from the random regression model.**

Age (years)	1	2	3	4	5	6	7	8	9	10	11	12	13	14	15	16	17	18
**1**	1																	
**2**	0.666 (0.008)	1																
**3**	0.543 (0.009)	0.658 (0.006)	1															
**4**	0.428 (0.011)	0.596 (0.007)	0.696 (0.005)	1														
**5**	0.34 (0.012)	0.533 (0.008)	0.668 (0.005)	0.744 (0.004)	1													
**6**	0.280 (0.013)	0.477 (0.009)	0.629 (0.006)	0.728 (0.004)	0.786 (0.003)	1												
**7**	0.241 (0.014)	0.429 (0.01)	0.586 (0.007)	0.698 (0.005)	0.774 (0.004)	0.821 (0.003)	1											
**8**	0.216 (0.014)	0.388 (0.011)	0.541 (0.008)	0.660 (0.005)	0.749 (0.004)	0.811 (0.003)	0.85 (0.003)	1										
**9**	0.202 (0.014)	0.353 (0.011)	0.499 (0.008)	0.62 (0.006)	0.716 (0.005)	0.790 (0.004)	0.842 (0.003)	0.874 (0.002)	1									
**10**	0.194 (0.014)	0.325 (0.012)	0.46 (0.009)	0.579 (0.007)	0.681 (0.005)	0.763 (0.004)	0.825 (0.003)	0.867 (0.002)	0.893 (0.002)	1								
**11**	0.192 (0.014)	0.304 (0.012)	0.427 (0.009)	0.543 (0.007)	0.646 (0.006)	0.734 (0.005)	0.804 (0.004)	0.854 (0.003)	0.887 (0.002)	0.906 (0.002)	1							
**12**	0.192 (0.014)	0.288 (0.012)	0.400 (0.010)	0.511 (0.008)	0.615 (0.007)	0.706 (0.005)	0.781 (0.004)	0.837 (0.003)	0.876 (0.002)	0.902 (0.002)	0.916 (0.001)	1						
**13**	0.195 (0.014)	0.277 (0.012)	0.379 (0.01)	0.485 (0.008)	0.588 (0.007)	0.68 (0.006)	0.757 (0.005)	0.818 (0.003)	0.861 (0.003)	0.891 (0.002)	0.911 (0.002)	0.922 (0.001)	1					
**14**	0.198 (0.014)	0.271 (0.012)	0.365 (0.010)	0.466 (0.009)	0.565 (0.008)	0.655 (0.007)	0.733 (0.005)	0.795 (0.004)	0.841 (0.003)	0.875 (0.002)	0.898 (0.002)	0.914 (0.002)	0.923 (0.001)	1				
**15**	0.200 (0.014)	0.269 (0.012)	0.357 (0.01)	0.451 (0.009)	0.544 (0.008)	0.631 (0.007)	0.705 (0.006)	0.766 (0.005)	0.812 (0.004)	0.847 (0.003)	0.873 (0.003)	0.893 (0.002)	0.909 (0.002)	0.920 (0.002)	1			
**16**	0.199 (0.015)	0.269 (0.012)	0.352 (0.01)	0.438 (0.01)	0.522 (0.009)	0.599 (0.008)	0.666 (0.007)	0.721 (0.006)	0.764 (0.005)	0.797 (0.004)	0.825 (0.004)	0.849 (0.004)	0.871 (0.003)	0.893 (0.003)	0.912 (0.002)	1		
**17**	0.188 (0.017)	0.266 (0.013)	0.345 (0.011)	0.419 (0.010)	0.487 (0.010)	0.548 (0.009)	0.599 (0.008)	0.642 (0.007)	0.676 (0.007)	0.704 (0.007)	0.73 (0.007)	0.757 (0.006)	0.786 (0.006)	0.82 (0.005)	0.859 (0.004)	0.897 (0.002)	1	
**18**	0.158 (0.021)	0.249 (0.015)	0.324 (0.012)	0.380 (0.012)	0.423 (0.012)	0.457 (0.011)	0.484 (0.011)	0.505 (0.011)	0.523 (0.010)	0.541 (0.010)	0.562 (0.010)	0.588 (0.010)	0.623 (0.009)	0.670 (0.008)	0.731 (0.007)	0.805 (0.005)	0.879 (0.003)	1

The lower triangle shows the genetic correlations between BMI across different ages (in years) as indicated in the column and row headers. Standard errors for each estimate are given in brackets.

Supplementary Table 7: Estimated fixed effects from the random regression model with and without adjusting for adult BMI PGS.

Model Term	Level	PGS-adjusted		*Unadjusted*	
		Fixed effects	SE	Fixed effects	SE
Intercept	-	2.8890	0.0019	2.9020	0.0019
Legendre polynomials	slope	0.1534	0.0013	0.1619	0.0014
	$slope^2$	0.0560	0.0009	0.0535	0.0009
	$slope^3$	-0.0326	0.0007	-0.0344	0.0007
Source(clinic/questionnaire)	questionnaire	-0.0170	0.0006	-0.0171	0.0006
Sex(male/female)	female	-0.0119	0.0026	-0.0111	0.0028
Interaction terms between sex and Legendre polynomials	female*slope	-0.0211	0.0019	-0.0207	0.0020
	female*$slope^2$	0.0095	0.0013	0.0093	0.0013
	female*$slope^3$	0.0046	0.0009	0.0044	0.0009
PGS	-	0.0990	0.0034		
Interaction terms between adult BMI and Legendre polynomials	PGS*slope	0.0657	0.0024		
	PGS*$slope^2$	-0.0189	0.0017		
	PGS*slope3	-0.0140	0.0012		

Source: measurement sources SE: standard errors of estimated fixed effect. PGS: polygenic score

Supplementary Table 8: Estimated variances and covariances for random effect terms in the random regression model for adult BMI PGS.

Unique Individual Variance-Covariance Matrix (K_i)				
	intercept	slope	slope^2	slope^3
intercept	0.0164 (0.0011)			
slope	0.0055 (0.0005)	0.0037 (0.0003)		
slope^2	-0.0028 (0.0003)	-0.0006 (0.0001)	0.0017 (0.0001)	
slope^3	-0.0014 (0.0001)	-0.0008 (0.0000)	0.0005 (0.0000)	0.0007 (0.0000)
**Additive Genetic Variance-Covariance Matrix (K_g)**				
	intercept	slope	slope^2	
intercept	0.0045 (0.0011)			
slope	0.0008 (0.0004)	0.0009 (0.0003)		
slope^2	-0.0008 (0.0003)	-0.0002 (0.0001)	0.0004 (0.0001)	
**Residual Variance**				
	0.0020 (0.0000)			

The variances (SE) are on the diagonals and the values below the diagonal are the covariances (SE)

Supplementary Table 9: **Estimated heritability of BMI at yearly intervals from one to 18 years of age from the random regression model conditioning on adult BMI PGS**

Age (years)	V_g	Var(V_g)	V_i	Var(V_i)	V_P	Var(V_P)	σ_e^2	Var(σ_e^2)	h^2_{SNP}	SE(h^2_{SNP})
**1**	1.98×10^{-3}	2.19×10^{-7}	3.89×10^{-3}	2.40×10^{-7}	7.88×10^{-3}	4.40×10^{-8}	2.02×10^{-3}	1.88×10^{-10}	0.251	0.059
**2**	1.64×10^{-3}	1.23×10^{-7}	2.91×10^{-3}	1.23×10^{-7}	6.56×10^{-3}	1.42×10^{-8}	2.02×10^{-3}	1.88×10^{-10}	0.249	0.053
**3**	1.57×10^{-3}	1.00×10^{-7}	3.09×10^{-3}	9.89×10^{-8}	6.68×10^{-3}	1.09×10^{-8}	2.02×10^{-3}	1.88×10^{-10}	0.235	0.047
**4**	1.70×10^{-3}	1.16×10^{-7}	3.81×10^{-3}	1.16×10^{-7}	7.52×10^{-3}	1.38×10^{-8}	2.02×10^{-3}	1.88×10^{-10}	0.225	0.045
**5**	1.95×10^{-3}	1.64×10^{-7}	4.79×10^{-3}	1.64×10^{-7}	8.77×10^{-3}	1.94×10^{-8}	2.02×10^{-3}	1.88×10^{-10}	0.223	0.046
**6**	2.28×10^{-3}	2.41×10^{-7}	5.99×10^{-3}	2.41×10^{-7}	1.03×10^{-2}	2.74×10^{-8}	2.02×10^{-3}	1.88×10^{-10}	0.222	0.047
**7**	2.63×10^{-3}	3.43×10^{-7}	7.46×10^{-3}	3.42×10^{-7}	1.21×10^{-2}	3.88×10^{-8}	2.02×10^{-3}	1.88×10^{-10}	0.217	0.048
**8**	2.97×10^{-3}	4.58×10^{-7}	9.21×10^{-3}	4.61×10^{-7}	1.42×10^{-2}	5.42×10^{-8}	2.02×10^{-3}	1.88×10^{-10}	0.209	0.047
**9**	3.25×10^{-3}	5.74×10^{-7}	1.12×10^{-2}	5.84×10^{-7}	1.65×10^{-2}	7.35×10^{-8}	2.02×10^{-3}	1.88×10^{-10}	0.197	0.046
**10**	3.47×10^{-3}	6.78×10^{-7}	1.34×10^{-2}	7.01×10^{-7}	1.89×10^{-2}	9.57×10^{-8}	2.02×10^{-3}	1.88×10^{-10}	0.184	0.043
**11**	3.60×10^{-3}	7.65×10^{-7}	1.55×10^{-2}	8.03×10^{-7}	2.11×10^{-2}	1.19×10^{-7}	2.02×10^{-3}	1.88×10^{-10}	0.171	0.041
**12**	3.65×10^{-3}	8.32×10^{-7}	1.72×10^{-2}	8.88×10^{-7}	2.28×10^{-2}	1.41×10^{-7}	2.02×10^{-3}	1.88×10^{-10}	0.160	0.040
**13**	3.63×10^{-3}	8.88×10^{-7}	1.82×10^{-2}	9.59×10^{-7}	2.38×10^{-2}	1.59×10^{-7}	2.02×10^{-3}	1.88×10^{-10}	0.152	0.039
**14**	3.54×10^{-3}	9.49×10^{-7}	1.84×10^{-2}	1.03×10^{-6}	2.39×10^{-2}	1.67×10^{-7}	2.02×10^{-3}	1.88×10^{-10}	0.148	0.041
**15**	3.42×10^{-3}	1.04×10^{-6}	1.76×10^{-2}	1.11×10^{-6}	2.31×10^{-2}	1.63×10^{-7}	2.02×10^{-3}	1.88×10^{-10}	0.148	0.044
**16**	3.29×10^{-3}	1.20×10^{-6}	1.64×10^{-2}	1.26×10^{-6}	2.18×10^{-2}	1.54×10^{-7}	2.02×10^{-3}	1.88×10^{-10}	0.151	0.050
**17**	3.21×10^{-3}	1.48×10^{-6}	1.57×10^{-2}	1.55×10^{-6}	2.09×10^{-2}	1.67×10^{-7}	2.02×10^{-3}	1.88×10^{-10}	0.154	0.058
**18**	3.23×10^{-3}	1.99×10^{-6}	1.70×10^{-2}	2.16×10^{-6}	2.23×10^{-2}	2.85×10^{-7}	2.02×10^{-3}	1.88×10^{-10}	0.145	0.063

V_g: estimated additive genetic variance; Var(V_g): estimated variance of the additive genetic variance component (V_g); V_i: estimated unique individual variance. Var(V_i): estimated variance of unique individual variance component; V_P: estimated phenotypic variance; Var(V_P): estimated variance of phenotypic variance; σ_e^2: estimated residual variance; Var(σ_e^2): estimated variance of residual variance component. h^2_{snp}: estimated SNP-heritability; SE(h^2_{snp}): standard error of SNP-heritability estimate.

Supplementary Table 10: Estimated heritability from LDSC of derived phenotypes from random regression model

Trait	h^2	*SE*	*Z*	h^2*_int*	h^2*_int_SE*
intercept	0.301	0.077	3.920	1.006	0.01
slope	0.324	0.075	4.340	0.999	0.01
slope2	0.167	0.067	2.490	1.001	0.01
PC1	0.301	0.076	3.960	1.006	0.01
PC2	0.359	0.080	4.480	0.991	0.01

Trait: Name of the trait (principal component-derived phenotype: PC1 or PC2), h2: Observed-scale SNP heritability estimated by LDSC, SE: Standard error of h2, Z: Z-score (h2 / SE), h2_int: LDSC heritability intercept (used to detect inflation due to confounding), h2_int_SE: Standard error of the heritability intercept.

Supplementary Table 11: Estimated genetic correlations from LDSC across derived phenotypes from random regression model

Trait 1	*Trait 2*	*rg*	*SE*	*Z*	*P*
intercept	slope	0.703	0.213	3.299	9.70E-04
intercept	slope2	-0.872	0.263	-3.316	9.15E-04
intercept	PC1	0.994	0.253	3.927	8.59E-05
intercept	PC2	0.152	0.170	0.896	3.70E-01
slope	slope2	-0.611	0.232	-2.630	8.53E-03
slope	PC1	0.773	0.217	3.557	3.75E-04
slope	PC2	-0.596	0.190	-3.131	1.74E-03
slope2	PC1	-0.874	0.263	-3.329	8.72E-04
slope2	PC2	-0.144	0.225	-0.641	5.22E-01
PC1	PC2	0.048	0.169	0.284	7.76E-01

Trait 1: First trait in the genetic correlation analysis, Trait 2: Second trait, rg: Genetic correlation between Trait 1 and Trait 2 estimated by LDSC, SE: Standard error of rg, Z: Z-score (rg / SE), P: P-value for rg. Statistical significance was assessed using a two-sided χ^2 test (1 degree of freedom). No multiple-comparison correction was applied.

Supplementary Table 12: **Comparison of the SNP-based heritability between BMI at different ages across early life in ALSPAC using the random regression model (RRM), cross-sectional genome-based restricted maximum likelihood (GREML) using GCTA, and MoBa (linkage disequilibrium score regression using cross-sectional data)**

Age in RRM	h^2_{SNP} (95% CI) RRM	Follow-up codes	Mean age (year)	h^2_{SNP} (95% CI) GCTA	P_gcta[1]	Age in Moba	h^2_{SNP} (95% CI) Moba	P_moba[1]
**1**	0.25 (0.13, 0.37)	Child health database 2	0.8	0.28 (0.14, 0.42)	0.75*	1	0.42 (0.34, 0.5)	0.014#
**1.5**	0.24 (0.12, 0.36)	Child health database 3	1.7	0.26 (0.12, 0.4)	NA	1.5	0.38 (0.32, 0.44)	0.04
**2**	0.24 (0.14, 0.34)	NA	NA	NA	NA	2	0.33 (0.25, 0.41)	0.14
**3**	0.23 (0.13, 0.33)	NA	NA	NA	NA	3	0.29 (0.23, 0.35)	0.34
**3.7**	0.28 (0.18, 0.38)	Child health database 4	3.7	0.17 (0.01, 0.33)	0.47	NA	NA	NA
**5**	0.25 (0.15, 0.35)	NA	NA	NA	NA	5	0.28 (0.22, 0.34)	0.62
**7**	0.28 (0.18, 0.38)	NA	NA	NA	NA	7	0.28 (0.2, 0.36)	0.89
**8**	0.28 (0.18, 0.38)	Focus@7	7.6	0.26 (0.14, 0.38)	0.82	8	0.33 (0.23, 0.43)	0.42
**10.7**	0.25 (0.17, 0.33)	Focus@10	10.7	0.28 (0.16, 0.4)	0.73	NA	NA	NA
**13.9**	0.25 (0.17, 0.33)	Teen Focus2	13.9	0.23 (0.09, 0.37)	0.82	NA	NA	NA
**15.5**	0.27 (0.17, 0.37)	Teen Focus3	15.5	0.26 (0.1, 0.42)	0.90	NA	NA	NA
**17.5**	0.30 (0.18, 0.42)	Teen Focus4	17.5	0.37 (0.17, 0.57)	0.57	NA	NA	NA

[1] P-value testing whether the estimated h2_SNP from GCTA or LDSC is different from estimates from RRM. Statistical significance was assessed by using a two-sided Z-test. No multiple-comparison correction was applied.
CI: confidence interval; RRM: random regression model
* Use 0.8-year estimate from GCTA to proxy the one year h2. # MoBA includes measurement under year one.

Supplementary Table 13: Estimated genetic correlations between multiple ages from cross-sectional genetic analyses in GCTA

Follow-up codes	chdb2 (0.8)	chdb3 (1.7)	chdb4 (3.7)	F7 (7.6)	F10 (10.7)	T2 (13.9)	T3 (15.5)	T4 (17.5)
**chdb2**	1 (0)							
**chdb3**	0.98 (0.11)	1 (0)						
**chdb4**	1 (0.20)	0.46 (0.39)	1 (0)					
**F7**	0.58 (0.15)	0.68 (0.15)	0.70 (0.19)	1 (0)				
**F10**	0.37 (0.25)	0.31 (0.25)	0.63 (0.29)	1 (0.03)	1 (0)			
**T2**	0.36 (0.50)	0.66 (0.32)	0.36 (0.50)	0.9 (0.07)	0.85 (0.07)	1 (0)		
**T3**	0.25 (0.24)	0.55 (0.20)	0.44 (0.26)	0.97 (0.07)	0.96 (0.08)	0.98 (0.03)	1 (0)	
**T4**	0.39 (0.20)	0.33 (0.25)	0.14 (0.37)	0.91 (0.1)	0.94 (0.07)	0.89 (0.06)	1 (0.06)	1 (0)

The genetic correlations for corresponding cross-sectional follow-ups (as indicated by the columns and rows) are presented in the lower triangle of the table, with standard errors enclosed in brackets. Follow-up codes are: chdb2: Child health database; chdb3: Child health database 3; F7: Focus@7; F10: Focus@10; T2: Teen Focus2; T3: Teen Focus3; T4: Teen Focus4. The average age (in years) in each code is shown in brackets in the header.

Supplementary Table 14: Estimated fixed effects from the random regression model with heterogenous errors.

Model Term	Level	Fixed effects	SE
Intercept	-	2.9020	0.0019
Legendre polynomials	slope	0.1624	0.0014
	$slope^2$	0.0531	0.0009
	$slope^3$	-0.0343	0.0007
Source (clinic/questionnaire)	questionnaire	-0.0159	0.0007
Sex (male/female)	female	-0.0111	0.0028
Interaction terms between sex and Legendre polynomials	female*slope	-0.0203	0.0020
	female*$slope^2$	0.0090	0.0014
	female*$slope^3$	0.0033	0.0010

Source: measurement sources SE: standard errors of estimated fixed effect.

Supplementary Table 15: Estimated variances and covariances for random effect terms in the random regression model heterogeneous errors

	**Unique Individual Variance-Covariance Matrix(K_i)**			
	intercept	slope	$slope^2$	$slope^3$
intercept	0.0168 (0.0013)			
slope	0.0053 (0.0005)	0.0036 (0.0003)		
$slope^2$	-0.0030 (0.0003)	-0.0007 (0.0001)	0.0018 (0.0001)	
$slope^3$	-0.0019 (0.0001)	-0.0010 (0.0000)	0.0006 (0.0000)	0.0008 (0.0000)
	**Additive Genetic Variance-Covariance Matrix(K_g)**			
	intercept	slope	$slope^2$	
intercept	0.0075 (0.0013)			
slope	0.0024 (0.0005)	0.0017 (0.0003)		
$slope^2$	-0.0012 (0.0003)	-0.0004 (0.0001)	0.0004 (0.0001)	
	**Residual Variances**			
Residual 1	0.0017 (0.0001)			
Residual 2	0.0010 (0.0000)			
Residual 3	0.0023 (0.0001)			
Residual 4	0.0016 (0.0001)			

Residual 5	0.0038 (0.0001)
Residual 6	0.0052 (0.0003)
Residual 7	0.0016 (0.0000)
Residual 8	0.0012 (0.0000)
Residual 9	0.0015 (0.0000)
Residual 10	0.0013 (0.0000)
Residual 11	0.0014 (0.0000)
Residual 12	0.0016 (0.0000)
Residual 13	0.0030 (0.0001)
Residual 14	0.0026 (0.0002)
Residual 15	0.0029 (0.0001)
Residual 16	0.00319 (0.0002)
Residual 17	0.00242 (0.0001)

The variances (SE) are on the diagonals and the values below the diagonal are the covariances (SE). The residual terms represent the estimated residuals of each yearly age bins ranging from 1 to 18 years, the number indicates the starting year of the corresponding age bin.

Supplementary Table 16: Estimated heritability of BMI at yearly intervals from one to 18 years of age from the random regression model with heterogeneous errors

Age (years)	V_g	Var(V_g)	V_i	Var(V_i)	V_P	Var(V_P)	σ_e^2	Var(σ_e^2)	h^2_{SNP}	SE(h^2_{SNP})
**1**	1.85×10^{-3}	2.20×10^{-7}	4.58×10^{-3}	2.49×10^{-7}	8.16×10^{-3}	5.96×10^{-8}	1.73×10^{-3}	4.25×10^{-9}	0.227	0.057
**2**	1.51×10^{-3}	1.25×10^{-7}	3.33×10^{-3}	1.27×10^{-7}	5.86×10^{-3}	1.86×10^{-8}	1.02×10^{-3}	2.11×10^{-9}	0.258	0.060
**3**	1.54×10^{-3}	1.06×10^{-7}	3.30×10^{-3}	1.05×10^{-7}	7.14×10^{-3}	1.58×10^{-8}	2.29×10^{-3}	3.69×10^{-9}	0.216	0.045
**4**	1.86×10^{-3}	1.30×10^{-7}	3.86×10^{-3}	1.28×10^{-7}	7.30×10^{-3}	2.39×10^{-8}	1.58×10^{-3}	8.26×10^{-9}	0.255	0.049
**5**	2.37×10^{-3}	1.92×10^{-7}	4.75×10^{-3}	1.87×10^{-7}	1.09×10^{-2}	3.37×10^{-8}	3.77×10^{-3}	1.11×10^{-8}	0.217	0.040
**6**	3.00×10^{-3}	2.91×10^{-7}	5.95×10^{-3}	2.82×10^{-7}	1.41×10^{-2}	1.01×10^{-7}	5.20×10^{-3}	6.86×10^{-8}	0.212	0.038
**7**	3.68×10^{-3}	4.21×10^{-7}	7.49×10^{-3}	4.09×10^{-7}	1.28×10^{-2}	4.98×10^{-8}	1.62×10^{-3}	1.90×10^{-9}	0.288	0.050
**8**	4.38×10^{-3}	5.73×10^{-7}	9.41×10^{-3}	5.60×10^{-7}	1.49×10^{-2}	6.99×10^{-8}	1.15×10^{-3}	1.08×10^{-9}	0.293	0.050
**9**	5.03×10^{-3}	7.31×10^{-7}	1.17×10^{-2}	7.20×10^{-7}	1.82×10^{-2}	9.72×10^{-8}	1.51×10^{-3}	1.36×10^{-9}	0.276	0.046
**10**	5.61×10^{-3}	8.79×10^{-7}	1.41×10^{-2}	8.78×10^{-7}	2.10×10^{-2}	1.29×10^{-7}	1.28×10^{-3}	8.94×10^{-10}	0.268	0.044
**11**	6.10×10^{-3}	1.01×10^{-6}	1.64×10^{-2}	1.02×10^{-6}	2.39×10^{-2}	1.64×10^{-7}	1.42×10^{-3}	1.41×10^{-9}	0.255	0.041
**12**	6.49×10^{-3}	1.12×10^{-6}	1.82×10^{-2}	1.15×10^{-6}	2.63×10^{-2}	1.99×10^{-7}	1.57×10^{-3}	1.96×10^{-9}	0.247	0.040
**13**	6.78×10^{-3}	1.21×10^{-6}	1.93×10^{-2}	1.25×10^{-6}	2.91×10^{-2}	2.27×10^{-7}	3.00×10^{-3}	3.71×10^{-9}	0.233	0.037
**14**	6.98×10^{-3}	1.31×10^{-6}	1.92×10^{-2}	1.35×10^{-6}	2.88×10^{-2}	2.61×10^{-7}	2.61×10^{-3}	2.43×10^{-8}	0.242	0.039
**15**	7.11×10^{-3}	1.44×10^{-6}	1.81×10^{-2}	1.47×10^{-6}	2.81×10^{-2}	2.40×10^{-7}	2.91×10^{-3}	7.90×10^{-9}	0.253	0.042
**16**	7.20×10^{-3}	1.66×10^{-6}	1.61×10^{-2}	1.64×10^{-6}	2.65×10^{-2}	2.41×10^{-7}	3.19×10^{-3}	2.60×10^{-8}	0.272	0.048
**17**	7.30×10^{-3}	2.02×10^{-6}	1.42×10^{-2}	1.98×10^{-6}	2.39×10^{-2}	2.38×10^{-7}	2.42×10^{-3}	1.58×10^{-8}	0.305	0.058
**18**	7.46×10^{-3}	2.63×10^{-6}	1.42×10^{-2}	2.67×10^{-6}	2.41×10^{-2}	3.68×10^{-7}	2.42×10^{-3}	1.58×10^{-8}	0.310	0.067

V_g: estimated additive genetic variance; Var(V_g): estimated variance of the additive genetic variance (V_g); V_i: estimated unique individual variance. Var(V_i): estimated variance of unique individual variance; V_P: estimated phenotypic variance; Var(V_P): estimated variance of phenotypic variance; σ_e^2: estimated residual variance; Var(σ_e^2): estimated variance of residual variance. h^2_{snp}: estimated SNP-heritability; SE(h^2_{snp}): standard error of SNP-heritability estimate.

Supplementary Table 17 Descriptive statistics of the final dataset in ALSPAC cohort used for RRM analysis

Categories	ALSPAC follow-up name	N (Male/Female)	Mean age (SD) [years]	Median of BMI (IQR) [kg/m2]
Nurse reports	Child health database 2	40 (13/27)	1.08 (0.07)	17.14 (2.26)
	Child health database 3	4454 (2219/2235)	1.7 (0.23)	16.8 (1.91)
	Child health database 4	4255 (2132/2123)	3.71 (0.2)	16.15 (1.75)
Child in Focus (CIF) *	CIF 12 months	730 (368/362)	1.03 (0.02)	17.84 (1.83)
	CIF 18 months	721 (372/349)	1.53 (0.03)	17.84 (1.83)
	CIF 25 months	683 (358/325)	2.08 (0.02)	17.09 (1.75)
	CIF 31 months	728 (376/352)	2.59 (0.02)	16.74 (1.73)
	CIF 37 months	723 (374/349)	3.08 (0.02)	16.61 (1.61)
	CIF 43 months	706 (367/339)	3.59 (0.02)	16.45 (1.55)
	CIF 49 months	706 (369/337)	4.07 (0.03)	16.36 (1.59)
	CIF 61 months	694 (361/333)	5.16 (0.07)	16.15 (1.55)
Clinic measurements	Focus@7	5350 (2710/2640)	7.56 (0.29)	15.81 (2.15)
	Focus@8	4651 (2332/2319)	8.68 (0.29)	16.62 (2.64)
	Focus@9	5181 (2566/2615)	9.91 (0.31)	17.02 (3.35)
	Focus@10	5228 (2581/2647)	10.68 (0.24)	17.5 (3.73)
	Focus@11	5072 (2498/2574)	11.78 (0.23)	18.26 (4.24)
	Teen Focus1	4735 (2316/2419)	12.84 (0.22)	19.05 (4.17)

	Teen Focus2	4404 (2151/2253)	13.88 (0.2)	19.63 (4.03)
	Teen Focus3	3885 (1867/2018)	15.48 (0.3)	20.71 (3.88)
	Teen Focus4	3052 (1376/1676)	17.44 (0.25)	21.81 (4.37)
Questionaries	My School Boy/Girl (KN)	2829 (1458/1371)	5.81 (0.09)	15.5 (2.06)
	My Daughter/Son Growing up (KP) round 1	803 (396/407)	6.37 (0.35)	15.63 (2.02)
	My Daughter/Son Growing up (KP) round 2	123 (64/59)	6.34 (0.41)	15.72 (1.87)
	Your Son/Daughter at 9 (KU) round 1	783 (397/386)	9.31 (0.75)	16.69 (3.23)
	Your Son/Daughter at 9 (KU) round 2	151 (65/86)	8.81 (0.7)	16.4 (2.18)
	Your Son/Daughter at 9 (KU) round 3	102 (50/52)	9.08 (0.71)	16.62 (3.85)
	Your Son/Daughter at 9 (KU) round 4	59 (27/32)	9.46 (0.54)	16.7 (3.35)
	My Teenage Son/Daughter (TA)	2706 (1343/1363)	13.18 (0.16)	19.07 (3.89)
	Your Son/Daughter at 16+ (TC)	2375 (1220/1155)	16.87 (0.36)	20.9 (3.35)

* Ten of the clinic follow-ups before 7 years of age were designed to focus on a 10% subset of individuals (termed the “Child in Focus”). N: sample size, SD: standard deviation, IQR: Interquartile range

Supplementary Notes

Supplementary Note 1: Validation and model checking

Cross-sectional analysis

We conducted a series of cross-section analyses to verify the results from our RRM. The SNP-based heritability from the cross-sectional genetic analyses in GCTA were consistent with the RRM, at 0.28 (SE=0.07) for 0.8 years to 0.37 (SE=0.10) at 17.5 years. However, the standard errors were larger in the cross-sectional analysis than in the RRM model, as expected (Supplementary Table 12). We also compared the cross-sectional genetic analyses from the MoBa cohort[1] to our RRM estimates, where the SNP-based heritability estimates were higher in MoBa than our data before 2 years of age (Supplementary Table 12). Similarly, the genetic correlations estimated using the cross-sectional data in GCTA were similar to the decreasing age-to-age genetic correlations pattern seen in the RRM analyses (Supplementary Table 13, Supplementary Figures 13 and 14). For instance, the 95% confidence interval around the estimated genetic correlation between Child health database 3 (mean age = 1.7 years) and Focus@7 (mean age = 7.6 years) (r_g=0.68, 95% CI = 0.39-0.97) overlapped with the 95% confidence interval around the RRM estimated genetic correlation between 2 and 8 years (r_g= 0.40, 95% CI = 0.16-0.64).

Heterogeneous error variance

Our model assumes homogeneous error variance, so we explored a model relaxing this assumption. Estimates of the unique individual and additive genetic variance using the RMM model with heterogeneous error variance were similar to those obtained from primary analysis. That is the 95% confidence intervals around the estimates overlap between the models (Supplementary Figure 15, Supplementary Tables 14-16). This indicates that while there may be some variation in the residuals over time, it does not appear to significantly impact our overall conclusions.

Validation of growth patterns

We obtained principal components from the genetic covariance matrix of childhood BMI from the COADTwins project for comparison to our results. PC1 in the CODATwins exhibited a

similar pattern from one to 18 years old to our findings. The variance explained by PC1 was roughly 89% in both datasets and we observed a similar shape of the eigenfunctions between the two studies (Supplementary Figure 16).

Supplementary Note 2: ASReml .as file for main analysis

```
!MP 16 !NO GRAPHICS !WORKSPACE 32 !RENAME

ALSPAC BMI repeated measures one year to 18 years

ID 6291   !A
age
sex 2 !A
weight
height
bmi
logbmi

grm_unrel_grm_ID_noheader_1_18y_3times.grm                                    !HINV
grm_unrel_grm_ID_noheader_1_18y_3times.txt
bmigrowth_ID_1_18y_3times.dat !skip 1 !MAXIT 150

log(bmi) ~ mu sex*leg(age,-3) !r leg(age,3).ide(ID) leg(age,2).giv(ID,1)

1 1 2                   #variance header line, no. R R G
0 0 IDV 0 !S2==1
leg(age,3).ide(ID) 2
leg(age,3) 0 US 0 !GP !+10
0.2928E-01
0.8908E-02  0.6640E-02
-0.5594E-02 -0.3050E-03  0.3351E-02
-0.7722E-05 -0.7399E-03  0.2433E-03  0.1227E-02
ide(ID)

leg(age,2).giv(ID,1) 2
leg(age,2) 0 US !GP !+6
0.002
0 0.001
0 0  0.001
ID 0 GIV1
```

Supplementary Note 3: R script for calculating 95% confidence intervals for the eigenvalues using numerical simulation

```
#loading estimated variances from .asr file
tmp1 = read.table("ALSPAC_RRM_g2_src_v3.asr",skip=94,nrow=17)

# residual
e = tmp1[1,5]

# unique individual effects
Ki = matrix(NA, nrow=4, ncol=4)
for (i in 1:10) {
  Ki[tmp1[(i+1),3],tmp1[(i+1),4]] = tmp1[(i+1),5]
  Ki[tmp1[(i+1),4],tmp1[(i+1),3]] = tmp1[(i+1),5]
}

# genetic effects
Kg = matrix(NA, nrow=3, ncol=3)
for (i in 1:6) {
  Kg[tmp1[(i+11),3],tmp1[(i+11),4]] = tmp1[(i+11),5]
  Kg[tmp1[(i+11),4],tmp1[(i+11),3]] = tmp1[(i+11),5]
}

# vvp estimated variance of variance components
# tmp2 = scan("ALSPAC_RRM_g2_v3.vvp",skip=1)
tmp2 = scan("ALSPAC_RRM_g2_src_v3.vvp",skip=1)
vvp = diag(0,nrow=17)
vvp[lower.tri(vvp,diag=T)] = tmp2
vvp[upper.tri(vvp,diag=T)] = tmp2
V = vvp[12:17,12:17] #estimated variance of the additive genetic variance component


##########################
# eigenvalue decomposition of Kg
##########################
Kg
# [,1]        [,2]        [,3]
# [1,]  0.00734918  0.002429220 -0.001162300
# [2,]  0.00242922  0.001728870 -0.000392931
# [3,] -0.00116230 -0.000392931  0.000400509
PCs = eigen(Kg)


# confidence limits for eigenvalues
library(ggplot2)
gHat = Kg[lower.tri(Kg,diag=T)]
# Cholesky decomposition
L = chol(V)
nvars = dim(V)[1]
```

```
simGcov = matrix(NA,nrow=6000,ncol=6)
simValues = matrix(NA,nrow=6000,ncol=3)
for (i in 1:6000) {
  simGcov[i,] = gHat + t(L) %*% matrix(rnorm(nvars))
  simG = matrix(NA,nrow=3, ncol=3)
  simG[lower.tri(simG,diag=T)] = simGcov[i,]
  simG[upper.tri(simG)] = simG[lower.tri(simG)]
  simValues[i,] = eigen(simG)$values
}

data1 = data.frame(values = c(simGcov), component = sort(rep(1:6,6000)))
data2 = data.frame(values = c(simValues), eigenValue = sort(rep(1:3,6000)))
est1 = data.frame(values = gHat, component = 1:6)
est2 = data.frame(values = PCs$values, eigenValue = 1:3)


# plot estimates with simulated ci
estimate = PCs$values
l.ci =  apply(simValues,2,quantile,0.025)
u.ci =  apply(simValues,2,quantile,0.975)


##calculate the 95% CI of variance explained by each PC
tot = rowSums(simValues)
simporp = matrix(NA,nrow=6000,ncol=3)
for (i in 1:6000) {simporp[i,] = simValues[i,]/tot[i]}
l.ci.porp =  apply(simporp,2,quantile,0.025)
u.ci.porp =  apply(simporp,2,quantile,0.975)
l.ci.porp
u.ci.porp

varExplained = PCs$values/sum(PCs$values)

data = data.frame(PC = paste0("PC",1:3),varExplained ,l.ci.porp,u.ci.porp)
plot <- ggplot(data) + geom_bar(aes(x=PC, y=varExplained), stat="identity", fill="skyblue")
+
  geom_errorbar(aes(x=PC, ymin=l.ci.porp , ymax=u.ci.porp), alpha=0.9, width = 0.1, size =
1.1, col="orange") +
  xlab("") + ylab("Variance Explained")
plot
```

Supplementary Note 4: ASReml .as file for adjusting for adult BMI PGS

```
!MP 16 !NO GRAPHICS !WORKSPACE 32 !RENAME

ALSPAC BMI repeated measures one year plus

ID 6291   !A
age
sex 2 !A
weight
height
bmi
source 2 !A
SCORESUM #PGS
logbmi


grm_unrel_grm_ID_noheader_1_18y_3times_v4.grm                    !HINV
grm_unrel_grm_ID_noheader_1_18y_3times_v4.txt
bmigrowth_ID_1_18y_3times_v4.dat !skip 1 !MAXIT 150 !ASUV


log(bmi) ~ mu sex*leg(age,-3) source SCORESUM*leg(age,-3) !r leg(age,3).ide(ID)
leg(age,2).giv(ID,1)

1 1 2              #variance header line, no. R R G
0 0 IDV 0 !S2==1
leg(age,3).ide(ID) 2
leg(age,3) 0 US 0 !GP !+10
  0.2278E-01
  0.7486E-02  0.5335E-02
 -0.3828E-09 -0.8910E-09  0.2167E-02
 -0.4635E-09 -0.5613E-03  0.6141E-03  0.7907E-03
ide(ID)

leg(age,2).giv(ID,1) 2
leg(age,2) 0 US !GP !+6
0
0 0
0 0 0
ID 0 GIV1
```

Supplementary Note 5: ASReml .as file for heterogeneity analysis

```
!MP 16 !NO GRAPHICS !WORKSPACE 32 !RENAME

ALSPAC BMI repeated measures one year plus

ID 6291   !A
age
sex 2 !A
weight
height
bmi
source 2 !A
logbmi

grm_unrel_grm_ID_noheader_1_18y_3times_v3.grm                                        !HINV
grm_unrel_grm_ID_noheader_1_18y_3times_v3.txt
bmigrowth_ID_1_18y_3times_v3.dat !skip 1 !MAXIT 150 !ASUV


log(bmi) ~ mu sex*leg(age,-3) source !r leg(age,3).ide(ID) leg(age,2).giv(ID,1) !f mv

17 1 2  # !STEP .01
5175 0  ID # !S2==1
2189 0  ID # !S2==1
5223 0  ID # !S2==1
1166 0  ID # !S2==1
3479 0  ID # !S2==1
969 0  ID # !S2==1
5190 0  ID # !S2==1
4563 0  ID # !S2==1
5040 0  ID # !S2==1
6241 0  ID # !S2==1
4903 0  ID # !S2==1
4503 0  ID # !S2==1
7069 0  ID # !S2==1
914 0  ID # !S2==1
3618 0  ID # !S2==1
1893 0  ID # !S2==1
3794 0  ID # !S2==1



leg(age,3).ide(ID) 2
leg(age,3) 0 US 0 !GP !+10
0.1650E-01
0.5330E-02  0.3516E-02
-0.2808E-02 -0.5955E-03  0.1684E-02
-0.1685E-02 -0.9866E-03  0.5489E-03  0.7707E-03
ide(ID)
```

```
leg(age,2).giv(ID,1) 2
leg(age,2) 0 US !GP !+6
0.7348E-02
0.2429E-02  0.1729E-02
-0.1162E-02 -0.3929E-03  0.4005E-03
ID 0 GIV1
```

Supplementary Note 6: Eigenvalue decomposition of genetic correlation matrix of BMI from the previously published COADTwins project

We performed eigenvalue decomposition using the genetic correlation matrix of BMI obtained from the previously published COADTwins project[2]. By doing this, we aimed to validate the pattern of genetic inheritance by estimating $\mathbf{K_g}$ using the reported genetic correlation matrix and heritability across different ages using the CODATwins project. This should give similar results to our decomposition of $\mathbf{K_g}$ given that the age ranges included in Silventoinen are similar to the current study.

Silventoinen *et al.* (2022) used Cholesky decomposition (i.e. multivariate AE model, i.e. a common statistical model in twin and adoption studies, which breaks down phenotypic variance into three parts: additive genetic [A] and unique environment [E] plus measurement error) on twin cohorts and reported correlation matrices of additive genetic components for both genders from 1 to 18 years[2]. We converted the correlation matrix to a covariance matrix (equivalent to $\mathbf{V_g}$; an 18 by 18 matrix) using the estimated standard deviations of the additive genetic component at each age. Since they did not provide estimates of heritability (h^2) or additive genetic variance, we approximated the genetic variance by using the phenotypic variance of BMI for each age and the previously reported heritability from CODATwins[3].

$$\begin{gathered}\widehat{v_g} = \widehat{v_p}\widehat{h^2} = \widehat{\mathrm{sd}_\mathrm{p}^2}\widehat{h^2} \\ \widehat{\mathrm{sd}_\mathrm{g}} = \sqrt{\widehat{v_g}} \\ \widehat{\mathrm{cov}_\mathrm{g}(\mathrm{t}_1,\mathrm{t}_2)} = \widehat{r_g(\mathrm{t}_1,\mathrm{t}_2)}\ \widehat{\mathrm{sd}_{\mathrm{g}1}}\ \widehat{\mathrm{sd}_{\mathrm{g}2}}\end{gathered} \qquad \text{Equation S1}$$

where $\widehat{v_g}$ is the estimated genetic variance of a given age, $\widehat{v_p}$ is the estimated phenotypic variance, $\widehat{h^2}$ is estimated heritability in twin study, $\widehat{sd_g}$ and $\widehat{sd_p}$ are estimated standard deviations of genetic and phenotypic variances, respectively, $\widehat{cov_g(\mathrm{t}_1,\mathrm{t}_2)}$ and $\widehat{r_g(\mathrm{t}_1,\mathrm{t}_2)}$ are estimated genetic covariance and correlation between ages t_1 and t_2, respectively. $\widehat{sd_{g1}}$ and $\widehat{sd_{g2}}$ are estimated standard deviations of genetic variances at time points t_1 and t_2, respectively. This covariance matrix was transformed into the covariance matrix of polynomials ($\mathbf{\Phi^+}$, approximation of $\mathbf{K_g}$, a 3 x 3 matrix) through the pseudo-inverse [4] of the $\mathbf{\Phi}$ matrix (Legendre coefficient matrix, 18 x 3 matrix, Equation 2 and 6 in main text[5]). The pseudo inverse matrix, $\mathbf{\Phi^+}$, can be calculated as follows.

$$\begin{gathered}\mathbf{\Phi^+} = (\mathbf{\Phi'\Phi})^{-1}\mathbf{\Phi'} \\ \hat{\mathbf{V}}_\mathbf{g} = \mathbf{\Phi\Phi^+\Phi'}\end{gathered} \qquad \text{Equation S2}$$

where $\hat{\mathbf{V}}_\mathbf{g}$ is of order $t \times t$, where t are ages across the BMI trajectory of interest for evaluation. Finally, we used the eigen() function in R to perform the eigenvalue decomposition on the polynomial covariance matrix and visualised the PCs for comparison to our results (**Supplementary Figure 13**).

References

1. Helgeland, O. *et al.* Characterization of the genetic architecture of infant and early childhood body mass index. *Nat Metab* **4**, 344-358 (2022).
2. Silventoinen, K. *et al.* Changing genetic architecture of body mass index from infancy to early adulthood: an individual based pooled analysis of 25 twin cohorts. *Int J Obes (Lond)* **46**, 1901-1909 (2022).
3. Silventoinen, K. *et al.* Genetic and environmental effects on body mass index from infancy to the onset of adulthood: an individual-based pooled analysis of 45 twin cohorts participating in the COllaborative project of Development of Anthropometrical measures in Twins (CODATwins) study. *Am J Clin Nutr* **104**, 371-9 (2016).
4. Penrose, R. A generalized inverse for matrices. *Mathematical Proceedings of the Cambridge Philosophical Society* **51**, 406-413 (1955).
5. Kirkpatrick, M., Lofsvold, D. & Bulmer, M. Analysis of the inheritance, selection and evolution of growth trajectories. *Genetics* **124**, 979-93 (1990).
