# Supplementary material for: Distinct genetic profiles influence body mass index between infancy and adolescence
Source: Nat Commun. 2026 Feb 19;17:1594. doi: 10.1038/s41467-026-69310-6 (PMC12920650; doi:10.1038/s41467-026-69310-6)
Supplement: Supplementary file 2 — Description of Additional Supplementary Files [file 41467_2026_69310_MOESM2_ESM.pdf]

**Description of Additional Supplementary Files**

**Supplementary Data 1.** Estimated genetic correlations from LDSC between derived phenotypes from the random regression model and complex traits and diseases

**Supplementary Data 2.** Summary of height and weight measurements excluded by growthcleanr with reasons, stratified by data source (clinic vs questionnaire)
