# Supplementary material for: Distinct genetic profiles influence body mass index between infancy and adolescence
Source: Nat Commun. 2026 Feb 19;17:1594. doi: 10.1038/s41467-026-69310-6 (PMC12920650; doi:10.1038/s41467-026-69310-6)
Supplement: Supplementary file 4 — Reporting Summary [file 41467_2026_69310_MOESM4_ESM.pdf]

nature portfolio

Corresponding author(s): Geng Wang and Nicole M Warrington (NCOMMS-24-52236A)

Last updated by author(s): Nov 11, 2025

Reporting Summary

Nature Portfolio wishes to improve the reproducibility of the work that we publish. This form provides structure for consistency and transparency in reporting. For further information on Nature Portfolio policies, see our Editorial Policies and the Editorial Policy Checklist.

Please do not complete any field with "not applicable" or n/a. Refer to the help text for what text to use if an item is not relevant to your study.
For final submission: please carefully check your responses for accuracy; you will not be able to make changes later.

Statistics

For all statistical analyses, confirm that the following items are present in the figure legend, table legend, main text, or Methods section.

n/a	Confirmed	
☐	☒	The exact sample size (*n*) for each experimental group/condition, given as a discrete number and unit of measurement
☐	☒	A statement on whether measurements were taken from distinct samples or whether the same sample was measured repeatedly
☐	☒	The statistical test(s) used AND whether they are one- or two-sided<br>*Only common tests should be described solely by name; describe more complex techniques in the Methods section.*
☐	☒	A description of all covariates tested
☐	☒	A description of any assumptions or corrections, such as tests of normality and adjustment for multiple comparisons
☐	☒	A full description of the statistical parameters including central tendency (e.g. means) or other basic estimates (e.g. regression coefficient) AND variation (e.g. standard deviation) or associated estimates of uncertainty (e.g. confidence intervals)
☐	☒	For null hypothesis testing, the test statistic (e.g. *F*, *t*, *r*) with confidence intervals, effect sizes, degrees of freedom and *P* value noted<br>*Give P values as exact values whenever suitable.*
☒	☐	For Bayesian analysis, information on the choice of priors and Markov chain Monte Carlo settings
☐	☒	For hierarchical and complex designs, identification of the appropriate level for tests and full reporting of outcomes
☐	☒	Estimates of effect sizes (e.g. Cohen's *d*, Pearson's *r*), indicating how they were calculated

Our web collection on statistics for biologists contains articles on many of the points above.

Software and code

Policy information about availability of computer code

Data collection	Data collection was performed centrally by Avon Longitudinal Study of Parents and Children (ALSPAC) study (https://www.bristol.ac.uk/alspac/). We provide detailed characteristics of ALSPAC participants and references to study descriptions in the Methods section of the manuscript.
Data analysis	We used published software, including ASReml (v4.2.1), Plink (v 1.9 and v 2.0), GCTA (v 1.94.1), and R (v 4.2.1; packages, e.g., 'growthcleanr') to analyse the data and produce plots for this manuscript. A full description of software used in this paper is provided in the Methods section, along with references to the relevant journal articles. The custom R and ASReml code used to perform the analyses in this study is available in the Supplementary Materials.

For manuscripts utilizing custom algorithms or software that are central to the research but not yet described in published literature, software must be made available to editors and reviewers. We strongly encourage code deposition in a community repository (e.g. GitHub). See the Nature Portfolio guidelines for submitting code & software for further information.

Data

Policy information about availability of data

All manuscripts must include a data availability statement. This statement should provide the following information, where applicable:

- Accession codes, unique identifiers, or web links for publicly available datasets
- A description of any restrictions on data availability
- For clinical datasets or third party data, please ensure that the statement adheres to our policy

The dataset supporting the current study has not been deposited in a public repository because of ALSPAC access policy but is available upon application via https://www.bristol.ac.uk/alspac/researchers/access/. GWAS summary statistics are available on the Program in Complex Trait Genomics website (https://cnsgenomics.com/content/data).

Research involving human participants, their data, or biological material

Policy information about studies with human participants or human data. See also policy information about sex, gender (identity/presentation), and sexual orientation and race, ethnicity and racism.

Reporting on sex and gender	The number of participants included at each follow-up, stratified by sex, is reported in Table 1. The random regression model included sex as a covariate.
Reporting on race, ethnicity, or other socially relevant groupings	In this study we report in terms of ethnicity. In ALSPAC, ethnicity was confirmed by principal components analysis.
Population characteristics	ALSPAC is a birth cohort where pregnant women resident in Avon, UK with expected dates of delivery between 1st April 1991 and 31st December 1992 were invited to take part in the study. Data from individuals aged between one and 18 years were included in the analyses, with male participants making up 50% of the individuals. Participants were not selected on the basis of disease status. All cohort specific details are provided in the manuscript.
Recruitment	See above for recruitment detail. Specific details for ALSPAC cohort are given in the methods and references.
Ethics oversight	Ethical approval for the study was obtained from the ALSPAC Ethics and Law Committee and the Local Research Ethics Committees.

Note that full information on the approval of the study protocol must also be provided in the manuscript.

Field-specific reporting

Please select the one below that is the best fit for your research. If you are not sure, read the appropriate sections before making your selection.

☒ Life sciences ☐ Behavioural & social sciences ☐ Ecological, evolutionary & environmental sciences

Life sciences study design

All studies must disclose on these points even when the disclosure is negative.

Sample size	The analysis comprised 6,291 ALSPAC participants.
Data exclusions	We excluded individuals who were part of a multiple birth (i.e., twins, triplets). We employed an automated data cleaning algorithm which excluded data observations of height and weight identified as biologically implausible (R package 'growthcleanr'). Individuals without genetic data were excluded. BMI measurements before one year or after 18 years were excluded.
Replication	The current study only includes data from ALSPAC, without external replication. However, we performed both internal validation using different methods (using cross-sectional models to validate estimates from the RRM) and detailed comparisons with previous studies (with the MoBa study and the CODATwins project).
Randomization	This study involves a population based cohort so randomization was not performed.
Blinding	No blinding is required because this study used data from ALSPAC, which was observational and had no specific interventions.

Reporting for specific materials, systems and methods

We require information from authors about some types of materials, experimental systems and methods used in many studies. Here, indicate whether each material, system or method listed is relevant to your study. If you are not sure if a list item applies to your research, read the appropriate section before selecting a response.

Materials & experimental systems

n/a	Involved in the study
☒	☐ Antibodies
☒	☐ Eukaryotic cell lines
☒	☐ Palaeontology and archaeology
☒	☐ Animals and other organisms
☒	☐ Clinical data
☒	☐ Dual use research of concern
☒	☐ Plants

Methods

n/a	Involved in the study
☒	☐ ChIP-seq
☒	☐ Flow cytometry
☒	☐ MRI-based neuroimaging

Plants

Seed stocks	*Report on the source of all seed stocks or other plant material used. If applicable, state the seed stock centre and catalogue number. If plant specimens were collected from the field, describe the collection location, date and sampling procedures.*
Novel plant genotypes	*Describe the methods by which all novel plant genotypes were produced. This includes those generated by transgenic approaches, gene editing, chemical/radiation-based mutagenesis and hybridization. For transgenic lines, describe the transformation method, the number of independent lines analyzed and the generation upon which experiments were performed. For gene-edited lines, describe the editor used, the endogenous sequence targeted for editing, the targeting guide RNA sequence (if applicable) and how the editor was applied.*
Authentication	*Describe any authentication procedures for each seed stock used or novel genotype generated. Describe any experiments used to assess the effect of a mutation and, where applicable, how potential secondary effects (e.g. second site T-DNA insertions, mosiacism, off-target gene editing) were examined.*

This checklist template is licensed under a Creative Commons Attribution 4.0 International License, which permits use, sharing, adaptation, distribution and reproduction in any medium or format, as long as you give appropriate credit to the original author(s) and the source, provide a link to the Creative Commons license, and indicate if changes were made. The images or other third party material in this article are included in the article's Creative Commons license, unless indicated otherwise in a credit line to the material. If material is not included in the article's Creative Commons license and your intended use is not permitted by statutory regulation or exceeds the permitted use, you will need to obtain permission directly from the copyright holder. To view a copy of this license, visit http://creativecommons.org/licenses/by/4.0/
